# Expression of human A53T alpha-synuclein without endogenous rat alpha-synuclein fails to elicit Parkinson’s disease-related phenotypes in a novel humanized rat model

**DOI:** 10.1371/journal.pone.0329823

**Published:** 2025-08-08

**Authors:** Nicole K. Polinski, Jukka Puoliväli, Leena Rauhala, Taina-Kaisa Stenius, Timo Bragge, Teija Parkkari, Anna-Maija Penttinen, Yi Chen, Omar S. Mabrouk, Kelly E. Glajch, Warren D. Hirst, Michael Perkinton, Terina N. Martinez, Mark A. Frasier

**Affiliations:** 1 Research Programs, The Michael J. Fox Foundation for Parkinson’s Research, New York, New York, United States of America; 2 Charles River Discovery Research Services, Charles River Laboratories, Kuopio, Finland; 3 Neurodegenerative Diseases Research Unit, Biogen, Cambridge, Massachusetts, United States of America; 4 Neuroscience BioPharmaceuticals R&D, AstraZeneca, Cambridge, United Kingdom; Indiana University, UNITED STATES OF AMERICA

## Abstract

Alpha-synuclein (aSyn) is linked to Parkinson’s disease (PD) through *SNCA* genetic mutations, phosphorylated aSyn in Lewy bodies and Lewy neurites, and most recently through evidence of aSyn aggregation in patient spinal fluid using the aSyn seed amplification assay. Therefore, understanding the biology of this protein and developing therapeutic interventions targeting pathological processing of aSyn are a key area of focus for novel treatments to slow or stop PD. Reliable preclinical models are imperative for these efforts. To this end, we developed a novel model using CRISPR/Cas9 to humanize the regions surrounding the naturally occurring threonine 53 amino acid in the Sprague Dawley rat to generate a humanized A53T aSyn rat model (aSyn A53T KI). We also generated an *Snca* knockout (aSyn KO) line to pair with the humanized A53T aSyn rat line to confirm that phenotypes were not due to loss of endogenous rat aSyn protein. A systematic phenotyping study was performed on these lines, assessing PD-related pathology and phenotypes at multiple timepoints. The aSyn KO rat line was profiled at 6 and 12 months of age, revealing successful aSyn protein knockout. The aSyn A53T KI model was profiled at 4, 8, 12, and 18 months of age for motor and non-motor phenotypes, nigrostriatal degeneration, and brain pathology. We confirmed the aSyn A53T KI rat expresses human aSyn while lacking endogenous rat aSyn. Motor function and non-motor function remain largely unaffected in this model, and no overt nigrostriatal degeneration or brain pathology are observed up to 18 months of age. Although the aSyn A53T KI rat lacks the ability to model PD pathology and phenotypes at baseline, it is an ideal model for investigating the impact of exogenous synuclein aggregates or environmental triggers on human aSyn in an *in vivo* model system.

## Introduction

Parkinson’s disease (PD) is the second most common neurodegenerative disorder, currently affecting approximately 1% of the population but predicted to increase in the coming years with an aging population [1]. The vast majority of PD cases are sporadic, with no known genetic link. Among the known genetic links are mutations in the *SNCA* gene that encodes the protein alpha-synuclein (aSyn), including multiplications or point mutations, such as A53T, A30P, E46K, H50Q, and G51D [2–8]. The link between aSyn and PD is also observed in cases without *SNCA* mutations, as aSyn is a major component in the pathological hallmarks of PD – Lewy bodies and Lewy neurites [9].

Given the clear genetic and etiological links between *SNCA/*aSyn and PD, many preclinical models used to study PD biology and preclinical therapeutic development research employ genetic, expression, or pathogenic changes in aSyn to model disease pathology. For *in vivo* rodent models, these include the use of viral vectors to overexpress wild-type (WT) or A53T mutant human aSyn [10], injection of pre-formed aSyn fibrils (PFF) to seed pathology in the endogenous protein [11], and transgenic or bacterial artificial chromosome (BAC)-transgenic overexpression of WT or mutant human aSyn [12,13]. These different model systems each have their benefits and limitations with regards to modeling PD pathology/phenotypes, construct validity, and practicality [14].

Briefly, the viral vector-based overexpression model induces nigrostriatal degeneration and behavioral phenotypes within a relatively quick time frame, but the reliance on high levels of aSyn overexpression to drive this pathology limits its relevance to the patient condition [15]. For the aSyn PFF model, dysfunction occurs as a result of pathology in endogenous aSyn without the need for supraphysiological levels of aSyn expression, which makes the pathogenic mechanism of this model more similar to what is seen in the patient condition. However, pathology in the model is heavily dependent on difficult aSyn PFF preparation and quality control steps, motor phenotypes of this model are not robust, and the long time frames needed for only moderate nigrostriatal degeneration can limit practicality [11,15]. Transgenic models benefit from having constitutive expression of human aSyn throughout the brain and other tissues, which enables modeling of the extra-nigral pathology and non-motor symptoms. Furthermore, transgenic models are more accessible as they do not require additional handling and intraparenchymal injections of viruses or PFFs. However, the drawback of these models is that the nigrostriatal system generally remains intact, phenotypes vary greatly based on the expression method and levels, and endogenous WT rodent aSyn is also present in most cases [12].

Recent efforts aimed to refine aSyn transgenic models to address some of these inherent drawbacks. This is important, as improved models with nigrostriatal and extranigral aSyn pathology and neurodegeneration in PD-relevant brain systems that result in motor and non-motor PD behavioral phenotypes would enable better translational modeling of PD biology and pathology. Such models would help to identify reliable PD biomarkers and develop novel therapeutics directed towards aSyn and its associated pathological mechanisms. Furthermore, expression of the human aSyn in the absence of the rodent homologous protein would enable more accurate testing of the many aSyn-targeting therapies—typically targeting human aSyn and not rodent aSyn—in a translational preclinical model *in vivo*. One of the first models attempting this was the SNCA-OVX model generated by the Wade-Martins lab that used BAC transgenic technology to express the complete human *SNCA* locus for near-physiological expression of human aSyn in mice lacking endogenous mouse aSyn protein [16]. Although this model does display striatal dopamine signaling deficits followed by nigral neuron loss and motor dysfunction, it lacks aSyn aggregation and phosphorylation at S129 in the substantia nigra, and aSyn is expressed at ~2-fold greater than endogenous levels [16]. More recently, a novel gene replacement technology was developed and used to replace the mouse *Snca* with 158kb of the human *SNCA* gene [17]. However, this model has not yet been phenotypically analyzed to evaluate the impact of the gene replacement on PD pathology and phenotypes.

Herein, we describe the generation and characterization of a novel Sprague Dawley rat line in which endogenous rat *Snca* gene was humanized using the CRISPR/Cas9 technology. This strategy was pursued to determine whether humanizing the amino acids surrounding the naturally occurring threonine at amino acid 53 in rat would result in PD-like pathology similar to what is seen in cases with the A53T mutation in humans. As this humanization prevented the expression of endogenous rat aSyn, we also created a separate aSyn knockout rat on the Sprague Dawley background through the introduction of a point mutation that generated a premature stop codon to determine whether any phenotypes in the humanized aSyn knock-in model were related to the loss of endogenous rat aSyn or introduction of humanized A53T aSyn. Homozygous knock-in rats were phenotyped with littermate controls across multiple time points, and their motor and non-motor phenotypes, nigrostriatal degeneration, synuclein pathology, and additional pathology markers were analyzed.

## Materials and methods

### Rat model generation

Two new rat lines were generated at SAGE Labs, Inc. (St. Louis, MO, USA) for this study. The first line is a humanized A53T aSyn rat on the Sprague Dawley background, in which CRISPR-Cas9 strategies were used to target the *Snca* region encoding amino acids 67–140 of aSyn which contains 6 amino acid differences, retaining the endogenous rat threonine at amino acid 53. To humanize 6 amino acid differences (specifically N87S, M100L, G103N, Y107A, S121D, and S122N) CRISPR was used to replace *Snca* amino acids 67–140 with a human *SNCA* cDNA (S1 Fig). This resulted in humanized A53T aSyn expression under the endogenous regulatory elements and promoter with a lack rat aSyn of expression. As a control, we also developed a rat aSyn KO line to ensure any phenotypes were not due to loss of endogenous rat aSyn. To generate this line, CRIPSR-Cas9 strategies were used in Sprague Dawley rats to insert a “T” base pair at position 17,446 in the genomic sequence of rat *Snca* to create a premature stop codon, which blocked rat aSyn protein expression. F1 heterozygous male and female rats of each line were bred to homozygosity at SAGE Labs. These lines are currently available to the research community through Taconic Biosciences (Rensselaer, NY, USA) as HsdSage:SD-*Snca*^*em1(SNCA-A53T)Sage*^ (22964, aSyn A53T KI) and HsdSage:SD-*Scna*^*em1Sage*^ (22966, aSyn KO).

### Breeding

All animal experiments were performed as specified in the license authorized by the National Animal Experiment Board of Finland (No. ESAVI/9296/04.10.07/2017) and according to the US National Institutes of Health (Bethesda, MD, USA) guidelines for the care and use of laboratory animals.

Animals used in this study were bred at Charles River Laboratories facility in Wilmington (MA, USA) to generate cohorts of homozygous rats with wild-type (WT) littermates. Two male homozygous aSyn KO and 3 male homozygous A53T aSyn KI rats were obtained from Horizon Discovery and bred with equal numbers of WT female Sprague Dawley rats from Horizon Discovery. This breeding scheme created a colony of heterozygous rats which were then bred to generate cohorts of homozygous, heterozygous, and WT littermates.

Homozygous and WT littermates were shipped to Charles River Discovery Research Services unit in Kuopio (Finland) at least 1 week before the experiments to allow for acclimatization. During the experiments, rats were housed in open top cages in a light-controlled environment (lights on at 07:00 am and off at 8:00 pm) at a temperature of 22 ± 1 °C and relative humidity of 40–70% with food (Teklad Global 2016, Envigo) and water provided *ad libitum*.

### Experimental scheme: KI rats

A cohort of 79 aSyn A53T KI rats (32 males and 47 females) and 82 WT littermates (34 males and 48 females) was used for the experiments elucidating effects of the A53T mutation. Animals were grouped according to age and genotype as described in Table 1.

**Table 1:** Experimental groups of KI rats and their WT counterparts.

Group	A53T KI		WT		Age at start (months)
	Male	Female	Male	Female	
KI1	9	10			4
KI2			9*	10	4
KI3	8	12			8
KI4			10	10**	8
KI5	10**	10**			12
KI6			5	16	12
KI7	5**	15****			18
KI8			10****	12*****	18

Four animals were subsequently excluded from data analyses owing to unclear QuantiGene (see QuantiGene Plex assay sub-section below). Several animals had to be culled for health reasons before experiments were completed. These reasons did not appear linked to the genotypes given their nature (e.g., growths, bloody discharge from uterus, compromised breathing, weight loss) and the fact that these welfare issues occurred at similar rates in WT and SNCA A53T KI rats. Rats culled before endpoint sampling due to welfare reasons are denoted with asterisks (*).

### Experimental scheme: KO rats

A cohort of 10 male and 9 female aSyn KO rats, as well as 10 male and 10 female WT littermate control rats were used for the experiments to determine the effect of endogenous rat aSyn absence. Animals were grouped according to age and genotype as described in Table 2.

**Table 2:** Experimental groups of KO rats and their WT counterparts.

Group	aSyn KO		WT		Age at start (months)
	Male	Female	Male	Female	
KO1	5	5			6
KO2			5	5	6
KO3	5	4			12
KO4			5	5	12

### Effects of A53T aSyn KI on rat behavior

To establish how expression of the A53T-mutated humanized aSyn influences rat locomotor function, we performed a range of behavioral tests in separate groups of aSyn A53T KI and litter-matched WT animals aged 4, 8, 12, and 18 months (Table 1).

#### Home cage activity.

Rats were subcutaneously implanted with radio-frequency (RF) identification chips under isoflurane anesthesia before testing to unambiguously identify individual animals in their home cages, which were placed on top of the receiver plates (Actual Analytics Ltd, Edinburgh, UK). Transmitting RF chips and receiver plates allowed analysis of home cage behavior of rats, including measurements of activity and preferred location in the home cage over a 72 h monitoring period.

#### Open field.

Spontaneous exploratory activity of aSyn A53T KI and WT littermate rats in the open field was examined using activity chambers (Med Associates, Inc., St Albans, VT, USA; 43.2 × 43.2 × 40 cm) equipped with infrared beams. Animals were tested in low-stress conditions with red light intensity lowered to approximately 10–30 lux. Rats were placed in the center of the chamber, and their behavior was recorded for 30 min and analyzed in 5 min intervals. Total distance moved, distance in the central area, average velocity, and rearing frequency were quantified.

#### Beam walk.

Integrity of the sensorimotor function was assessed by analyzing how rats traverse a 160 cm horizontal tapered (square) beam that had underhanging ledges on each side to permit foot faults without falling [18]. A bright light was placed above the platform at the start point to motivate rats to traverse the beam. The end of the beam was connected to a black escape box (20.5 cm × 25 cm × 25 cm). Prior to the testing day, animals were pretrained to traverse the beam in three sessions over 3 preceding days. Rat beam walking was videotaped and the slip ratio (the number of slips/number of total steps) was calculated. Steps onto the ledge were scored as a slip. The mean of three successive trials was used for statistical analyses.

#### Fine motor skills and gait.

A fully automated kinematic analysis system (MotoRater, TSE Systems GmbH, Bad Homburg, Germany) was used to analyze fine motor skills and gait of rats. The experimental set-up included a brightly illuminated Plexiglas corridor (153 × 5 × 10 cm) and a high-speed recording camera (300 fps) situated under that corridor [19]. MotoRater mirrors positioned at specific angles allowed simultaneous capture of rat movements from the left, right and ventral aspects by using Motion Capture and Analysis software (Simi Reality Motion Systems, Unterschleißheim, Germany). On the day of testing, the rats were marked in appropriate points of body, such as joints of limbs and parts of the tail, with a non-toxic paint to facilitate detection of movements. Rat locomotion parameters and gait characteristics were extracted from video recordings of five to six complete strides along the corridor. A stride was defined as movement between two consecutive left hindlimb floor contacts. Raw marker trajectory data were further analyzed using a custom analysis system. During the parameter determination procedure, the initial ground contacts and the onsets of the swing phases for each limb were detected first and then, strides were determined. The analyzed parameters included: 1) general gait pattern parameters (stride time and speed, step width, stance and swing time during a stride, interlimb coordination), 2) body posture and balance (toe clearance, iliac crest and hip height, hind limb protraction and retraction, tail position and movement), and 3) fine motor skills (swing speed during a stride, jerk metric during swing phase, angle ranges and deviations of different joints, vertical and horizontal head movement). Strides were excluded from analysis if the animal stopped for any reason, or if the stride was unusually slow (slower than 50% of the median speed). The correlation structure of the parameters was assessed using principal component analysis (PCA). An overall gait analysis score based on the differences in PC scores between aSyn A53T KI and WT rats was established and determined as described earlier [19]. The basis of the score was the discriminant vector that characterized effects of the A53T-mutated SNCA on gait parameters. The overall gait score for each rat was obtained by projecting normalized parameter data onto the discriminant vector.

### Effect of A53T aSyn KI on gastrointestinal tract motility

We assessed stool frequency on two successive days for 1 h each time. Each rat was removed from its home cage and placed in a clean, clear plastic cage without food or water for 1 h. Stools were collected immediately after expulsion and placed in sealed tubes. The total stools were weighed to provide a wet weight, then dried overnight at 65 °C and weighed again to provide a dry weight. In addition, number of stools was calculated.

For bead expulsion time measurements, rats were fasted for 12 h prior to the experiment. After that, a single 3 mm colored plastic bead was inserted under brief isoflurane anesthesia into the distal colon (3 cm past the anus) with a plastic rod. Animals were observed for 1 h to measure time until bead expulsion. If the bead was not expelled during the entire observation period, time of 60 min was used for statistical comparisons.

### Endpoint sampling

At the endpoint, rats were terminally anesthetized by pentobarbital (60 mg/kg, Mebunat, Orion Pharma, Espoo, Finland). Following cardiac puncture and terminal blood sampling, rats were transcardially perfused with heparinized saline.

#### Brain tissue collection.

Right brain hemispheres of all aSyn KO rats and right brain hemispheres of half of aSyn A53T KI and litter-matched WT rats were dissected into the cortex, striatum, hippocampus, substantia nigra pars compacta (SNpc), and cerebellum for gene expression analyses. The right hemisphere samples, with the exception of the striatal samples in the aSyn KO (for HPLC; see below), of all aSyn KO, half of aSyn A53T KI, and their litter-matched WT rats, were collected as 5–10 mg punches, immersed in 5–10-fold greater volume of RNAlater, and stored at +4 °C overnight. Thereafter, RNAlater was removed and samples were stored at −80 °C until used for RNA transcript analyses using QuantiGene branched DNA (bDNA) technology (ThermoFisher Scientific, Waltham, MA, USA). Right striatum samples of all aSyn KO, half of aSyn A53T KI, and their litter-matched WT rats were weighed, fresh frozen in liquid nitrogen, and stored at −80 °C to be used for HPLC analysis.

Left brain hemisphere samples (cortex, striatum, hippocampus, substantia nigra pars compacta and cerebellum) from all aSyn KO rats, half of aSyn A53T KI rats, and their WT littermates were weighed, fresh frozen in liquid nitrogen, and stored at −80 °C to be used for western blotting. The left brain hemispheres of the remaining half of the aSyn A53T KI and litter-matched WT rats were dissected out in toto, post-fixed in 4% paraformaldehyde solution for 24 h, cryoprotected in a 30% sucrose solution in 0.1 M phosphate buffer (PB) for 72 h (until sunk), and frozen in liquid nitrogen for immunohistochemistry analysis.

#### Gut tissue collection.

In half of the aSyn A53T KI and litter-matched WT rats, the gut was dissected out, and 3 cm samples of the duodenum (6 cm anal to the pyloric sphincter) and proximal colon (the first 6 cm distal to the cecum) were excised, cut open longitudinally, and carefully washed with phosphate buffer saline (PBS) to remove intestinal contents. Then, the samples were rolled into a package in the opposite direction to the intestinal structure (i.e., against the longitudinal axis) to form a roll-like structure. The package was tied with threads prior to fixation in 4% PFA in 0.1 M PB for a minimum of 24 h. The samples were then transferred to a 30% sucrose solution (in 0.1 M PB) at +4 °C for 48–72 h. After fixation and cryoprotection, the threads were removed, and the samples were frozen in liquid nitrogen and placed in foil on dry ice to maintain the original organ structure. The samples were stored at −80 °C until sectioning. Before sectioning, the collected duodenum and colon rolls were placed in separate Peel-A-Way® Disposable Embedding Molds (each sample from the same rat in its own mold) and embedded in Tissue-Tek Embedding Medium O.C.T. compound.

### Immunohistochemistry

#### Sectioning.

The fixed, cryoprotected, and O.C.T.-embedded left hemisphere samples were sectioned with a cryostat in the coronal plane at an interval of 200 µm. In each cohort, the brains were cut into 6–8 full series of 20 µm thick cryosections, each containing representative sections of the cortex, striatum, hippocampus, SNpc, and cerebellum. Sections were collected on Superfrost glass slides using the following coordinates: slide A, 0.4 mm to −0.4 mm from bregma (striatum and cortex; five sections), slide B, −3.2 mm to −4.0 mm from bregma (hippocampus; five sections); slides C and D, −4.6 mm to −6.2 mm from bregma (SNpc; nine sections); and slide E: −9.2 mm to −10.0 mm from bregma (cerebellum; five sections).

Gut rolls were sectioned coronally (against the axis of the roll) with a cryostat. The duodenum and colon were sectioned separately into 20 µm thick cryosections. In total, three series at an interval of 200 µm were collected, with four sections per series. One series was used for the total aSyn staining, one for the phosphorylated aSyn staining, and one was reserved for piloting and negative controls.

#### Staining.

*TH staining and stereology:* Brain sections containing SNpc were stained for tyrosine hydroxylase (TH) and counterstained with cresyl fast violet as follows. Cryosections were air-dried for 30 min and washed three times with 1x PBS, pH 7.4. Endogenous peroxidase was blocked by incubating the sections in a 0.3% H_2_O_2_ solution in methanol for 30 minutes. Then the sections were washed with a 0.3% solution of Triton X-100 in PBS (PBST; pH 7.4) and blocked with a 10% normal goat serum (NGS) solution in PBST for 30 min at room temperature. Sections were then washed with a 5% NGS solution in PBST for 5 min and incubated with the same solution containing a polyclonal rabbit antibody against TH (#AB152, Merck Millipore, Espoo, Finland) at 1:1000 dilution in 5% NGS solution in PBST overnight at room temperature. The following day, the sections were washed three times with PBS containing 5% NGS and incubated with the secondary biotinylated goat anti-rabbit antibody (Vector Laboratories, Inc., Newark, CA, USA) diluted 1:500 in the same solution for 2 h at room temperature. Then, the sections were washed three times with PBS containing 5% NGS and incubated with the avidin/biotin-based peroxidase system (Vector Laboratories, Inc., Newark, CA, USA) diluted in PBS (5 µL of reagent A + 5 µL of reagent B + 1,000 µL of PBS) for 120 min at room temperature. Sections were then washed three times with PBS, and chromogen was developed with ImmPact 3,3′-diaminobenzidine (Vector Laboratories, Inc., Newark, CA, USA) for 1 min, Visual examination until desired color was reached. The reaction was stopped by placing the sections in ddH2O three times.

Cresyl fast violet counterstaining was performed for 4 min, and the dye was washed with ddH_2_O twice for 90 s. Sections were treated with an increasing ethanol series of (70%, 95%, and 100%), cleared with Roticlear, mounted with Rotimount, and coverslipped for semiquantitative stereological analysis. The total numbers of TH-positive cells in the SNpc were estimated using the optical fractionator method [20]. The cells of interest were counted manually, with the region of interest for each section being separately delineated using Stereo Investigator 11 software (MicroBrightField, Williston, VT, USA). Usually, about 10 − 20 counting frames per section were analyzed. The Stereo Investigator 11 algorithm was used to take into account the region of interest area (ROI), number of counted TH-positive neurons, section thickness, and interval between the sections to calculate an estimation of the TH-total positive cell count within the ROI.

*Phospho-Tau AT8 brain staining:* Brain cryosections were air-dried for 30 min and then washed three times with 1X TBS (pH 7.4). Antigen retrieval was performed in 1X Citrate buffer, pH 6.0 for 5 minutes at 80° C. Sections were washed once in 1X TBS (pH 7.4). Then, the sections were permeabilized using 0.3% Triton X-100 in TBS (pH 7.4) (TBST, pH 7.4), blocked with a 10% NGS solution in TBST for 30 min at room temperature. Sections were washed once with 5% NGS in TBST. Then, the sections were incubated overnight at room temperature with mouse monoclonal antibody against Phospho-Tau (Ser202, Thr205) Monoclonal Antibody (AT8) (MN1020, Thermo Fisher Scientific, Waltham, MA, USA) at 1:500 dilution in 5% NGS in TBST. The following day, sections were washed three times with 1X TBS (pH 7.4) and incubated in the secondary goat anti-mouse AF568 antibody (A21124, Molecular Probes, Eugene, Oregon, USA) diluted 1:500 in 5% NGS in 1x TBS for 2 h at room temperature. Then, the sections were washed three times with 1X TBS (pH 7.4), twice with double distilled H_2_O, twice with 70% EtOH and counterstained with 0.3% Sudan black B in 70% EtOH to quench autofluorescence. Finally, the sections were rinsed three times with 70% EtOH, washed three times in ddH2O, mounted, and coverslipped using ProLong Gold with DAPI. Positive control tissue from P301S tau transgenic mice was used to confirm staining success (S2A Fig).

*Iba-1 brain staining:* Brain cryosections were air-dried for 30 min and then washed once with 1X PBS (pH 7.4). Antigen retrieval was performed in 1X Citrate buffer, pH 6.0 for 5 minutes at 80° C. Sections were washed once in 1X PBS (pH 7.4). Then, the sections were permeabilized using PBST (pH 7.4) blocked with a 10% NGS solution in PBST for 30 min at room temperature. Sections were washed once with 1% NGS in PBST then incubated overnight at room temperature with rabbit polyclonal antibody against ionized calcium-binding adapter molecule1 (Iba-1,019-1974A, Fujifilm WAKO, Osaka, Japan) at 1:5000 dilution in 1% NGS in 1x PBST. The following day, sections were washed three times with PBST and incubated in the secondary goat anti-rabbit AF568 antibody (A11036, Molecular Probes, Eugene, Oregon, USA) diluted 1:500 in 1% NGS in 1x PBST for 2 h at room temperature. The sections were then washed three times with 1X PBS (pH 7.4), twice with double distilled H_2_O, twice with 70% EtOH and counterstained with 0.3% Sudan black B in 70% EtOH to quench autofluorescence. Finally, the sections were rinsed three times with 70% EtOH, washed three times in double distilled H_2_O, mounted, and coverslipped using ProLong Gold with DAPI.

*GFAP brain staining:* Brain cryosections were air-dried for 30 min and then washed three times with 1X PBS (pH 7.4). Then, the sections were permeabilized using PBST (pH 7.4) blocked with a 10% NGS solution in PBST for 20 min at room temperature. Sections were washed once with 1% NGS in PBST. Then, the sections were incubated overnight at room temperature with rabbit polyclonal antibody against total glial fibrillary acidic protein (GFAP, #Z0334, Agilent DAKO, Santa Clara, CA, USA) at 1:5000 dilution in 1% NGS in PBST. The following day, sections were washed three times with 1X PBS (pH 7.4) and incubated in the secondary goat anti-rabbit AF568 antibody (A11036, Molecular Probes, Eugene, Oregon, USA) diluted 1:500 in 1% NGS in PBST for 2 h at room temperature. The sections were then washed three times with 1X PBS (pH 7.4), twice with ddH_2_O twice with 70% EtOH and counterstained with 0.3% Sudan black B in 70% EtOH to quench autofluorescence. Finally, the sections were rinsed three times with 70% EtOH, washed three times in ddH2O, mounted, and coverslipped using ProLong Gold with DAPI.

*pS129 aSyn brain staining:* Brain cryosections were air-dried for 30 min and then washed three times with 1X TBS (pH 7.4). Then, the sections were permeabilized using PBST (pH 7.4) blocked with a 10% NGS solution in PBST for 30 min at room temperature. Sections were again washed with a 1% NGS solution in TBST for 5 min. The sections were then incubated overnight at room temperature with rabbit monoclonal primary antibody against phosphorylated (pS129) aSyn (#ab51253; Abcam, Cambridge, UK) at 1:500 dilution in 1% NGS in PBST. The following day, sections were washed three times with TBS (pH 7.4) and incubated in the secondary goat anti-rabbit AF568 antibody (A11036, Molecular Probes, Eugene, Oregon, USA) diluted 1:500 in 1% NGS in TBST for 2 h at room temperature. Then, the sections were washed three times with TBS (pH 7.4), twice with ddH_2_O twice with 70% EtOH and counterstained with 0.3% Sudan black B in 70% EtOH to quench autofluorescence. Finally, the sections were rinsed three times with 70% EtOH, washed three times in ddH2O, mounted, and coverslipped using ProLong Gold with DAPI. Positive control tissue from LRRK2 G2019S KI mice injected with an adeno-associated virus to overexpress human A53T aSyn was used to confirm staining success (S2B Fig).

*Total aSyn brain staining:* Brain cryosections were air-dried for 30 min and then washed three times with 1X TBS (pH 7.4). Then, the sections were permeabilized using PBST (pH 7.4) blocked with a 10% NGS solution in TBST (pH 7.4) for 20 min at room temperature. The sections were then incubated overnight at room temperature with rabbit polyclonal antibody against total aSyn (#2628S, Cell Signaling Technology, Beverly, MA, USA) 1:200 dilution in 1% NGS in 1x TBST. The following day, sections were washed three times with 1X TBS pH 7.4 and incubated in the secondary goat anti-rabbit AF568 antibody (A11036, Molecular Probes, Eugene, Oregon, USA) diluted 1:500 in 1% NGS in 1x PBST for 2 h at room temperature, Then, the sections were washed three times with 1X TBS pH 7.4, twice with 70% EtOH and counterstained with 0.3% Sudan black B in 70% ethanol to quench autofluorescence. Finally, the sections were rinsed three times with 70% EtOH, washed three times in ddH2O, mounted, and coverslipped using ProLong Gold with DAPI.

*pS129 aSyn gut staining:* Gut cryosections were air-dried for 30 min and then washed three times with 1X TBS (pH 7.4). Then, the sections were permeabilized using PBST (pH 7.4) (TBST, pH 7.4) for 20 minutes blocked with a 10% NGS solution in PBST (pH 7.4) for 30 min at room temperature. Sections were again washed with a 1% NGS solution in PBST for 5 min. The sections were then incubated overnight at room temperature with rabbit monoclonal primary antibody against phosphorylated (pS129) aSyn (#ab51253; Abcam, Cambridge, UK) at 1:500 dilution in 1% NGS in 1x PBST. The following day, sections were washed three times with TBST and incubated in the secondary goat anti-rabbit AF568 antibody (A11036, Molecular Probes, Eugene, Oregon, USA) diluted 1:500 in 1% NGS in TBST for 2 h at room temperature, Then, the sections were washed three times with 1X TBS (pH 7.4), twice with 70% EtOH and counterstained with 0.3% Sudan black B in 70% ethanol to quench autofluorescence. Finally, the sections were rinsed three times with 70% EtOH, washed three times in ddH2O, mounted, and coverslipped using ProLong Gold with DAPI.

*Total aSyn gut staining* Gut cryosections were air-dried for 45 min and then washed three times with 1X PBS (pH 7.4). Then, the sections were permeabilized using PBST (pH 7.4) blocked with a 10% NGS solution in PBST pH 7.4 for 45 min at room temperature. The sections were then incubated overnight at room temperature with rabbit polyclonal antibody against total aSyn (#2628S, Cell Signaling Technology, Beverly, MA, USA) 1:500 dilution in 1% NGS in 1x PBST. The following day, sections were washed three times with 1X PBS (pH 7.4) and incubated in the secondary goat anti-rabbit AF568 antibody (A11036, Molecular Probes, Eugene, Oregon, USA) diluted 1:500 in 1% NGS in 1x PBST for 2 h at room temperature, Then, the sections were washed three times with 1X PBS (pH 7.4), twice with 70% EtOH and counterstained with 0.3% Sudan black B in 70% ethanol to quench autofluorescence. Finally, the sections were rinsed three times with 70% EtOH, washed three times in ddH2O, mounted, and coverslipped using ProLong Gold with DAPI.

### Expression of the endogenous rat *Snca* and A53T-mutated humanized *SNCA* genes

#### Tissue processing for QuantiGene Plex assay.

All punch biopsies of the cerebral cortex, striatum, hippocampus, substantia nigra, and cerebellum (approximately 5–10 mg total tissue weight each) were homogenized using the Working Homogenizing Solution prepared as per the manufacturer’s protocol in the QuantiGene Sample Processing Kit for Tissues (Thermo Fisher Scientific) as follows. After thawing, any excess RNAlater was carefully removed, and the tissue piece was homogenized using Ø5mm steel beads and a TissueLyser II device (Qiagen, Hilden, Germany). The homogenization volumes were adjusted to tissue weight (300 µL of the homogenizing solution + 3 µL of proteinase K per every 5 mg of tissue). The homogenates were incubated at 65°C for 30 min with mixing in between, after which the samples were centrifuged twice. The clear RNA-containing supernatants from the second step were collected and stored at −80°C in two aliquots, where possible, to avoid repeated freeze-thaw cycles.

#### QuantiGene Plex assay.

Expression levels of the target genes were determined by the branched DNA assay using custom-prepared QuantiGene Plex sets and a QuantiGene Plex Assay Kit according to the manufacturer’s instructions (Thermo Fisher Scientific/Invitrogen). Small pilots were run before the actual assays to determine the optimal sample input: a 1:5–1:8 dilution of the original tissue lysate was ultimately used for all of the samples (depending on the tissue) to generate signals well above the limit of detection but not exceeding the linear range of the assay.

In aSyn KO rats and their WT littermates, expression levels of the endogenous *Snca* gene were examined in the cortex, hippocampus, cerebellum, and substantia nigra pars compacta. In aSyn A53T KI rats and their WT littermates, expression levels of the endogenous rat *Snca* gene and humanized A53T-mutated *SNCA* gene were determined in the cortex, striatum, hippocampus, cerebellum, and substantia nigra pars compacta. Signals associated with expression of endogenous rat *Snca* and humanized *SNCA* genes were normalized to the geometric mean of expression levels of three housekeeping genes (*Hmbs*, *Ppib*, and *Gapdh*). All targets analyzed in separate regions of the right brain hemisphere using the QuantiGene Plex assay are detailed in Table 3.

**Table 3:** Genes for which mRNA expression levels were determined using the QuantiGene (bDNA) method.

Target symbol	Target name	NCBI reference sequence	Samples
Rat *Snca*, upstream, (MGC105443; bases 3–309)	Rat aSyn	NM_019169	KO, WT_KO_
Rat *Snca*, downstream, (MGC105443; bases 564–1082)*	Rat aSyn	NM_019169	KO, WT_KO_ KI, WT_KI_
Human *SNCA* (bases 95–548)^#^	Human aSyn KI	NM_000345 (custom probe set ID: GS03265)	KI, WT_KI_
*Hmbs*	Rat hydroxymethylbilane synthase	NM_013168	KO, WT_KO_ KI, WT_KI_
*Ppib*	Rat peptidylprolyl isomerase B	NM_022536	KO, WT_KO_ KI, WT_KI_
*Gapdh*	Rat glyceraldehyde-3-phosphate dehydrogenase	NM_017008	KO, WT_KO_ KI, WT_KI_

* Outside of the KI region in the last exons.

^#^ *SNCA* knock-in (NM_019169, bases 1–327 + NM_000345, bases 424–648) designed to produce signal only if the knock-in is present (captured human portion only).

As there was little previous characterization of the aSyn KO rat, two different probe sets for the *Snca* mRNA transcript were tested. aSyn KO rats had one base pair insertion in the *Snca* gene, which produces a premature stop codon in exon 4. Consequently, *Snca* probes were designed to lie both upstream and downstream of exon 4 to take into account the possibility that the KO genetic modification would result in the differential degradation of the transcript along the length of the mature mRNA. The design, implemented by Thermo Fisher Scientific as a highly customized assay, involved the probe sets binding to the same physical transcript, which would then shear (post-hybridization to the probe sets) in between the hybridized regions. Each piece (upstream: bases 3–309; downstream: bases 564–1082) then produced a unique signal that was measured independently.

The final QuantiGene panel IDs used were M20011701 for the aSyn A53T KI rats and their WT littermates, and M19073105 for the aSyn KO rats and their WT littermates.

#### QuantiGene Plex assay data processing.

The non-normalized (median) fluorescence intensity (MFI) values reflecting mRNA expression levels of respective genes were obtained using either a Bio-Plex 200 reader (BioRad, Hercules, CA, USA) or MAGPIX System (Luminex Corporation, Austin, TX, USA) and converted to normalized gene expression values as detailed below. For assay quality control, the limit of detection was defined as the average MFI of the background control wells plus three standard deviations calculated for each assay plate. The signals below the thus defined limit of detection were not removed from analyses because such signals were in groups that were expected to generate low signal, and exclusion would have decreased the effective number of replicates. Thus, all values were used for statistical analyses, even though the exact values that were close to background may not have been precisely quantified (e.g., the expression level of the humanized A53T *SNCA* in WT animals). Additionally, after removing potential (technical) outliers, the MFI values were corrected to the baseline (each analyte separately) by subtracting the average MFI in the background wells. These net MFI values were averaged and then normalized by dividing each target gene signal (net MFI) by the geometric mean of the housekeeping genes (averaged and with background subtracted).

#### Western blot.

For tissue homogenization, fractionation, and western blot analysis, we applied a slightly modified version of previously described protocol [21]. Tissue was homogenized using a FastPrep®-24 device in eight volumes (v/w) of the solution containing 50 mM HEPES-KOH, 1% Triton X-100, 750 mM NaCl, 5 mM EDTA (pH 7.6) supplemented with Complete Mini protease inhibitor cocktail and PhosSTOP phosphatase inhibitor cocktail (Roche Diagnostics). For fractionation, homogenates were normalized for the protein content and centrifuged at 16,100 g for 45 min at 4 °C. The collected supernatant comprised the soluble fraction, whereas the pellet was resuspended in four volumes of a solution containing 50 mM HEPES-NaOH and 1% SDS (pH = 7.6) and represented the insoluble fraction. Lysates normalized for the total protein concentration were then subjected to SDS-PAGE on Bis-Tris NuPAGE 4–12% gradient gels under reducing conditions and transferred to polyvinylidene difluoride membranes. The membranes were fixed with fresh 0.4% paraformaldehyde in PBS for 30 min and then incubated with the 4B12 antibody against human aSyn (MA-90346, Thermo Fisher Scientific), syn-1 antibody against total aSyn (clone 42, BD Biosciences, Franklin Lakes, NJ, USA), or C4 antibody against actin (MAB1501, MIllipore). Bound antibodies were detected and quantified by LICOR infrared imaging system and ImageStudio Lite 5.2.5 software, respectively. Whole blot images can be found in S3 Fig.

### HPLC

Samples were derivatized using a slightly modified version of a previously reported method [22]. Briefly, 20ul of diluted homogenate was added to a round bottom 96 well plate. 10 ul of 100mM Sodium Carbonate was added to each sample and agitated by pipetting up and down for 5 seconds. Then, 10 ul of 2% Benzoyl Chloride was added to each sample and again pipetted up and down for 5 seconds. Finally, 10 ul of an internal standard mixture (containing 13C-benzoyl chloride labeled dopamine, HVA, DOPAC) was added to the sample and pipetted up and down. Samples were then loaded onto a Shimadzu Sil-20 AC autosampler which injected samples onto a Waters BEH C18 reverse phase column (2 mm ID x 5 cm long). Samples were separated and eluted over 10 minutes, and an AB Sciex 6500 triple quadrupole mass spectrometer was used to detect benzoylated dopamine (466 - > 105), benzoylated HVA (304 - > 105), and benzoylated DOPAC (394 - > 105). Concentrations were calculated using a standard curve and the light:heavy internal standard ratio.

### Statistical analysis

All data are presented as the mean ± standard deviation (SD) or as box-whisker plots. For the analysis of gene and protein expression, data were pooled across sexes, whereas physiological and behavioral parameters were analyzed in males and females separately. Data were analyzed by the two-way analysis of variance (ANOVA) with genotype and age as factors. Data from consecutive measurements in the same animals were analyzed by averaging data from time bins and performing two-way repeated measures ANOVA with age and genotype as within-subject and between-subject factors, respectively. When factor interaction effects were statistically significant, Holm–Šídák’s multiple comparisons test to explore differences between genotypes at individual levels of the time or age factor. Data on striatal monoamine levels were analyzed using mixed-effects model (REML) with genotype and age as independent factors. Raw data can be found on Zenodo in the MJFF Data Repository collection (https://doi.org/10.5281/zenodo.16109481).

## Results

### Confirmation of rat aSyn knockout and human A53T aSyn knockin at mRNA and protein levels in mutant rats

**Fig 1:**
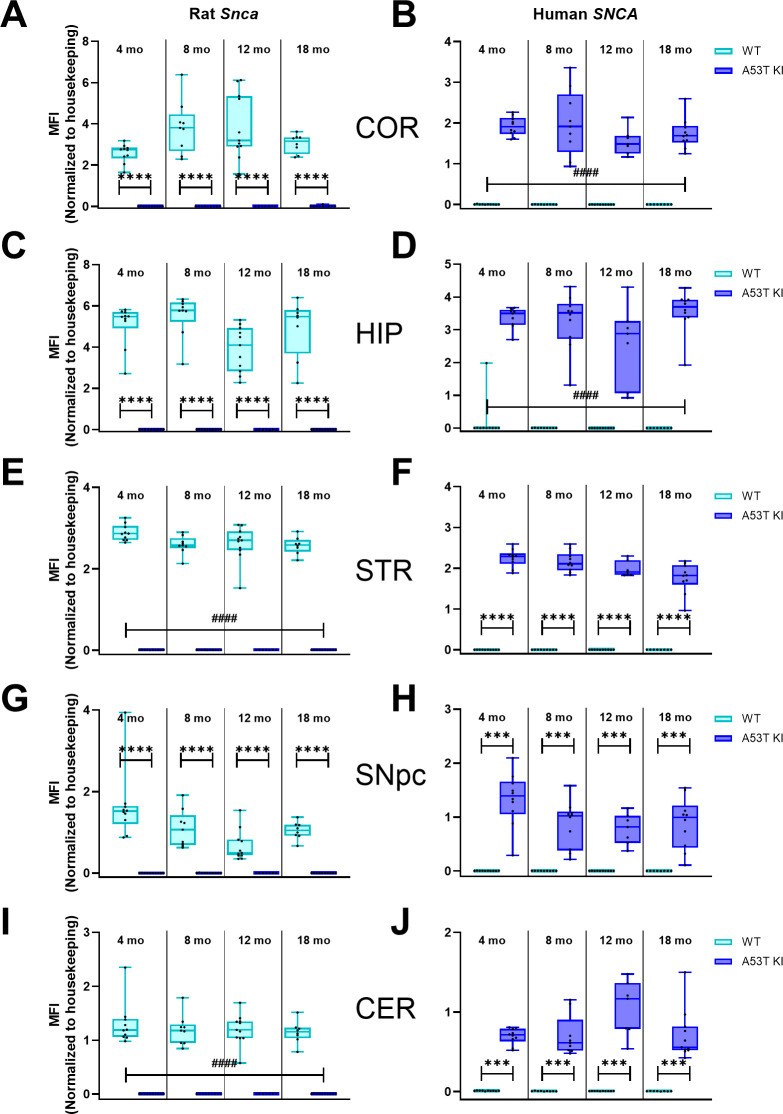
The aSyn A53T KI rat line expresses A53T-mutated humanized *SNCA* gene without rat *Snca* in the brain. Transcript levels (designated as median fluorescence intensity (MFI) of endogenous rat *Snca* and mutated humanized *SNCA* genes were quantified in the right brain hemisphere samples using the QuantiGene Plex assay and normalized to the geometric mean of mRNA expression levels of housekeeping genes *Hmbs*, *Ppib*, and **G*apdh* in the cortex (A, B), hippocampus (C, D), striatum (E, F), substantia nigra (G, H), and cerebellum (I, J). Data are presented as box-whisker plots. Data were analyzed using ordinary two-way ANOVA with genotype and age as independent factors. If the genotype × age effect was significant, pairwise comparisons within the same age group were done by the Holm–Šídák’s multiple comparisons test as follows: ***P < 0.001 ****P < 0.0001. If the factor interaction effect was not significant, main genotype effect was illustrated as follows: ^####^*P* < 0.0001.

**Fig 2:**
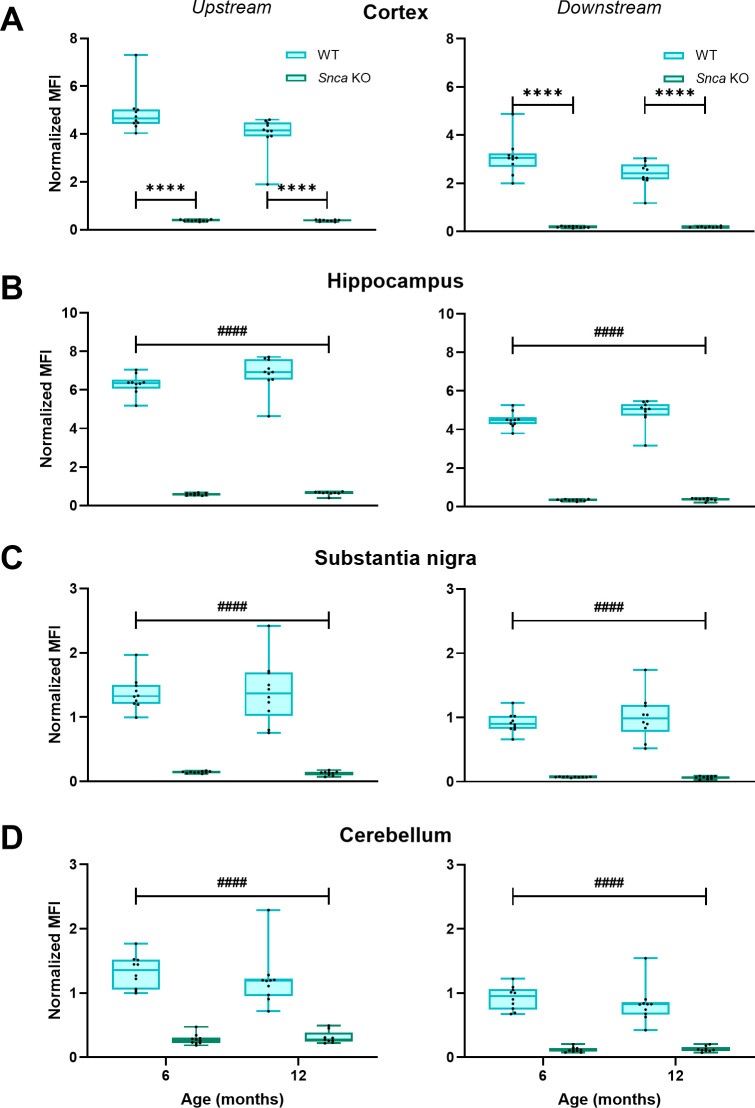
Confirmation of successful rat *Snca* depletion in the aSyn KO rat line. The QuantiGene Plex assay that utilized upstream and downstream probe sets for rat *Snca* showed specific signals (designated as Normalized MFI) only in samples from WT rats but not in samples from aSyn KO animals in the cortex (A), hippocampus (B), substantia nigra (C) and cerebellum (D). Data were analyzed using ordinary two-way ANOVA with genotype and age as independent factors. If the genotype × age effect was significant, pairwise comparisons within the same age group were done by the Holm–Šídák’s multiple comparisons test as follows: ****P < 0.0001. If the factor interaction effect was not significant, main genotype effect was illustrated as follows: ^####^*P* < 0.0001.

To confirm the genetic modifications, we first measured mRNA levels of rat *Snca* and human A53T *SNCA* mRNA. We confirmed expression of the human A53T *SCNA* mRNA in multiple brain regions of aSyn A53T KI rats and absence of this mRNA in the brain of WT animals (Fig 1B, D, F, H, J) in cohorts of all four different ages. In turn, probe sets selective for the endogenous rat *Snca* gene generated a positive signal only in samples from WT rats, demonstrating successful ablation of endogenous rat *Snca* expression through humanization of the gene (Fig 1A, C, E, G, I). Successful knockout of rat *Snca* was confirmed using upstream and downstream probe sets to the single base pair insertion in the aSyn KO rats (Fig 2).

**Fig 3:**
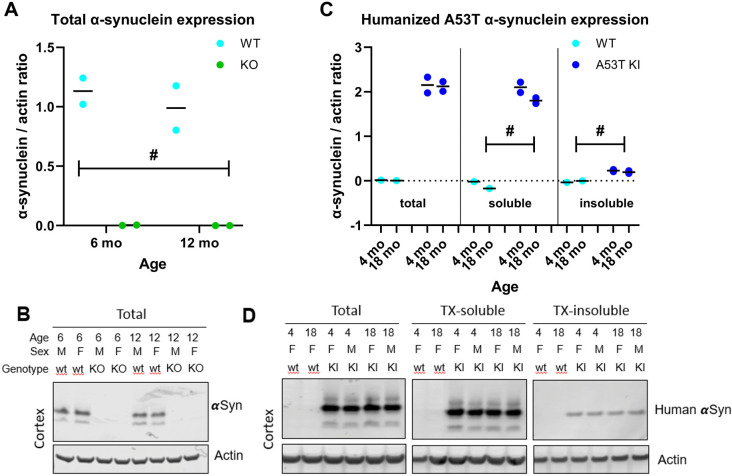
Confirmed loss of aSyn protein the aSyn KO line and expression of human aSyn protein in the aSyn A53T KI line. (A) Relative expression of rat aSyn in the cortex of aSyn KO and associated WT rats. (B) Western blot image of total aSyn and actin western blot in cortex of aSyn KO and WT rats. (C) Relative expression of human aSyn in the cortex of aSyn A53T KI and associated WT rats. (D) Western blot image of total aSyn, triton-soluble aSyn, and triton-insoluble aSyn in cortex of aSyn A53T KI and WT rats. Horizontal bars indicate the median value. Data were analyzed using two-way repeated measures ANOVA with genotype and age as independent factors. Main genotype effect was illustrated as follows: ^#^*P* < 0.05.

Next, we confirmed successful genetic modifications at the protein level. Western blots were performed in cortical extracts of aSyn KO rats and WT littermates using a mouse anti-aSyn antibody (clone 42, BD Biosciences), showing successful knockout of the aSyn protein in the aSyn KO rat line (Fig 3A, B). To measure the expression of human aSyn in the aSyn A53T KI rat, we performed western blotting of cortical extracts using a monoclonal antibody specific to human aSyn (clone 4B12; Thermo Fisher Scientific), showing selective expression of the humanized aSyn in KI rats (Fig 3C, D). Together, these observations confirm specificity of gene targeting and reliable brain mRNA and protein expression or loss thereof in the aSyn A53T KI and aSyn KO lines, respectively.

### Gastrointestinal function and locomotor behavior are not impaired in the aSyn A53T KI model

Gross observations and body weight measurements were normal in the aSyn A53T KI rats. For a more granular look at the impact of the humanized A53T aSyn mutation on the rats, we performed detailed gastrointestinal and motor function analyses.

**Fig 4:**
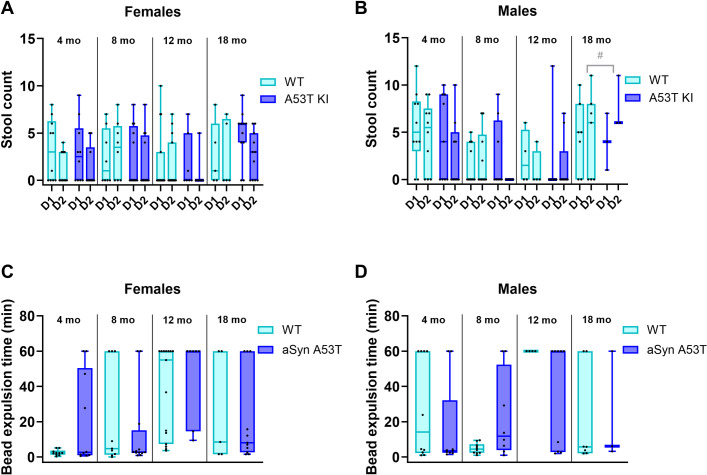
Gastrointestinal motility is generally unchanged in aSyn A53T KI rats. (A, B) Stool counts in female and male aSyn A53T KI and WT rats of different ages. (C, D) Bead expulsion times in female and male aSyn A53T KI and WT rats of different ages. Data in A–D are presented as box-whisker plots. Data were analyzed using ordinary two-way ANOVA with genotype and age as independent factors (Stool count averaged over D1 and D2).

Gastrointestinal motility parameters, including stool counts and bead expulsion times, were largely similar in aSyn A53T KI and WT rats (Fig 4A–D).

**Fig 5:**
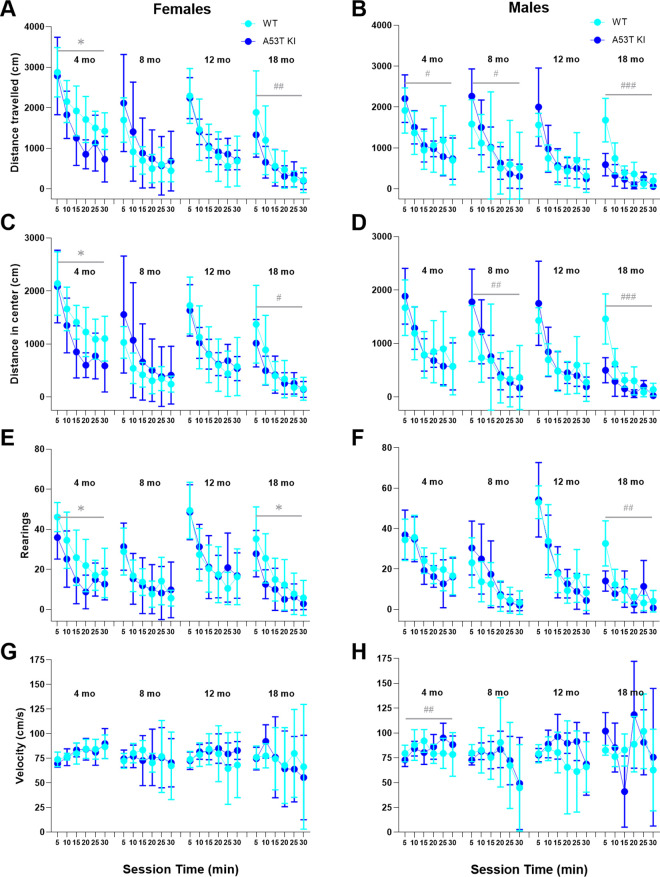
Open field locomotion is not robustly affected in aSyn A53T KI rats. Open field measures of total distance travelled (A, B), distance travelled in the center (C, D), number of rearings (E, F), and velocity (G, H). Data are presented as the mean ± S.D (n = 3–15). Statistical analysis was conducted from sums of distances traveled during time bins except for velocity average of velocity during time bins was used. Data were analyzed using ordinary two-way ANOVA with genotype and age as independent factors.

Assessment of behavior of aSyn A53T KI rats in the open field did not reveal any significant differences between the groups on the four parameters in females and males (Fig 5). Two-way ANOVA with genotype and age as independent factors was conducted using sums of distances traveled or number of rearings over five-minutes time bins, except for the velocity, average velocity over the time bins was used.

**Fig 6:**
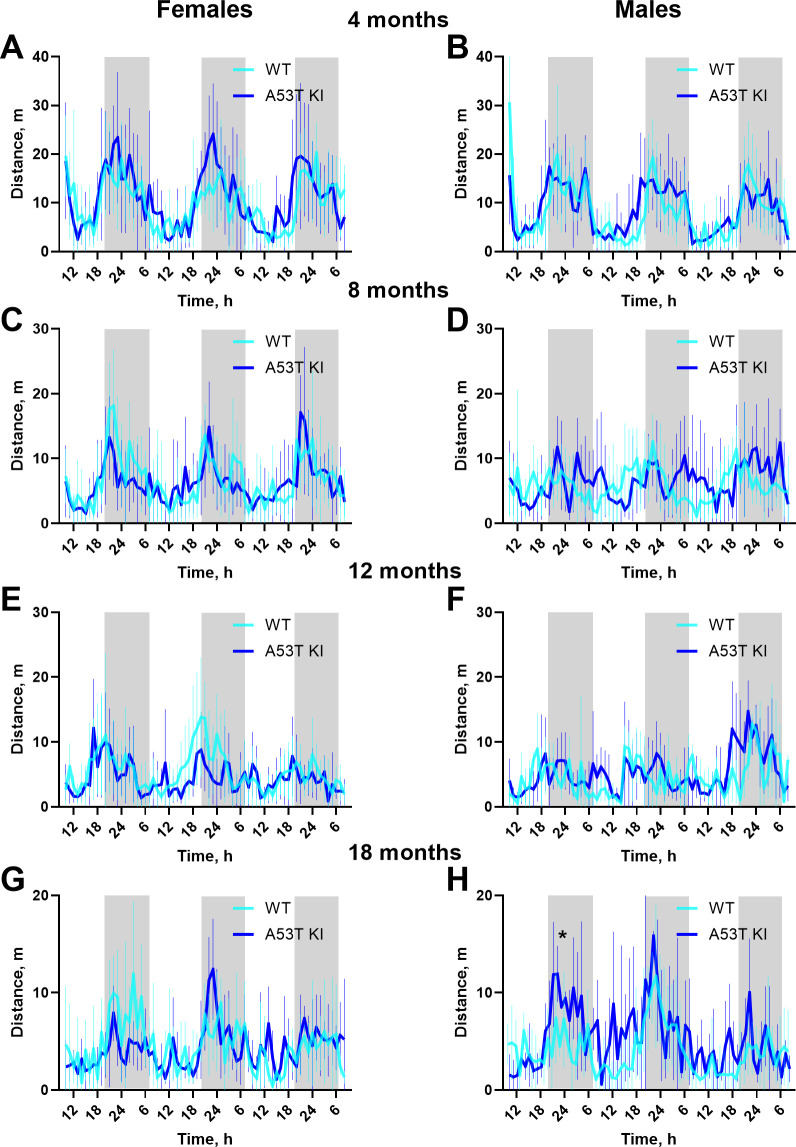
Distance travelled in home cage by A53T KI and WT rats. Distance travelled in home cage by female (A, C, E, G) and male (B, D, F, H) A53T KI and WT rats. Data are presented as the mean ± S.D. (*n* = 8–10 per each hourly point). Gray boxes indicate periods with lights switched off. For statistical analysis the distance travelled during the measurement period within age month were averaged and analyzed using ordinary two-way ANOVA.

**Fig 7:**
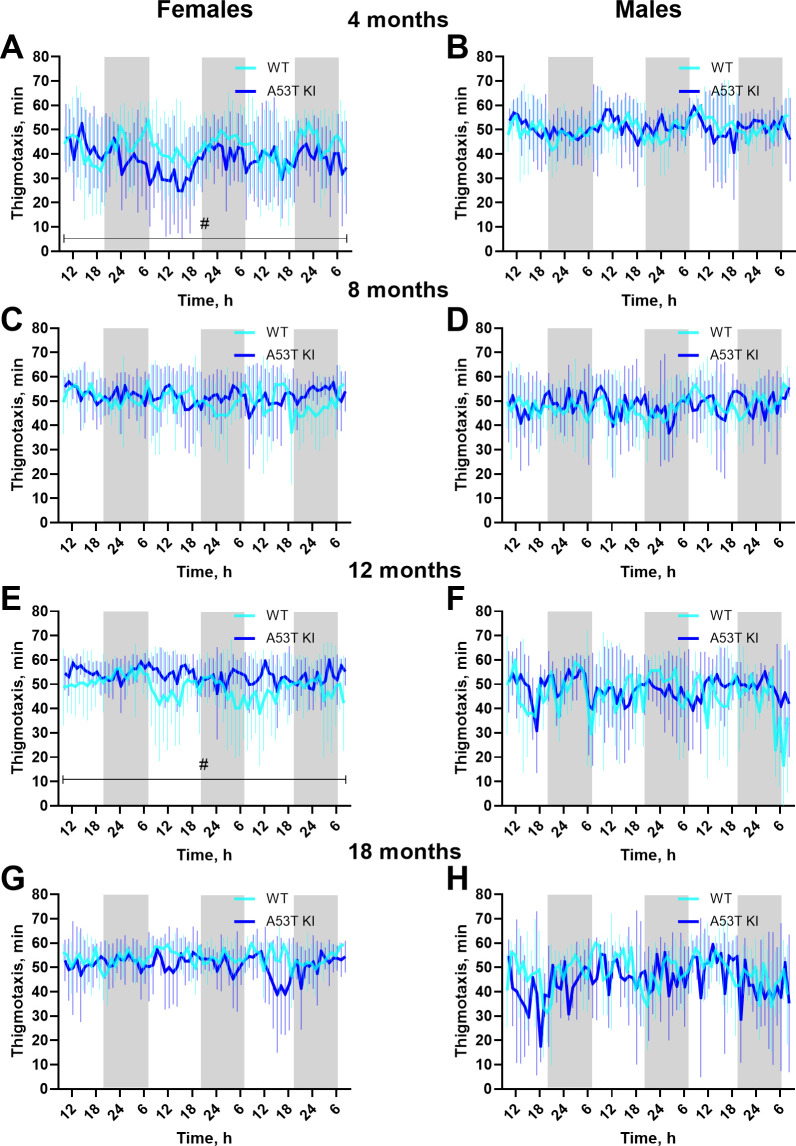
Thigmotactic behavior in home cage of A53T KI and WT rats. Thigmotactic behavior expressed as time spent near home cage walls in female (A, C, E, G) and male (B, D, F, H) A53T KI and WT rats. Data are presented as the mean ± S.D. (*n* = 8–10 per each hourly point). Gray boxes indicate periods with lights switched off. Averaged data were analyzed using ordinary two-way ANOVA with genotype and age as independent factors. If the genotype × age effect was significant, pairwise comparisons within the same age group were done by the Holm–Šídák’s multiple comparisons test as follows: *P < 0.05.

Next, we moved to monitoring animals in their home cages as this enables a less disruptive assessment of their behavior and readouts during sleep/wake cycles. We examined WT and aSyn A53T KI rats in continuous 72 h recordings, analyzing primarily distance travelled and duration of thigmotactic behavior (i.e., tendency to remain close to cage walls) in 1 h bins (Figs 6 and 7). Statistical analysis was performed on time bin averaged data using genotype and age as independent factors. The aSyn A53T KI rats demonstrated similar average distance travelled than WT in both males and females (*P* > 0.05, two-way ANOVA interaction and main effect of Genotype) (Fig 7).

A statistically significant interaction effect was observed in thigmotaxis in females (F _(__3, 69__)_ = 5.467, *P* = 0.0020) with aSyn A53T KI animals showing less thigmotaxis at 4 months of age (*P* = 0.0437, Holm-Šidák’s test) and more thigmotaxis at 12 months of age (*P* = 0.0306) (Fig 7). Given the inconsistent direction of change and minimal differences, these thigmotaxis differences do not seem physiologically relevant.

**Fig 8:**
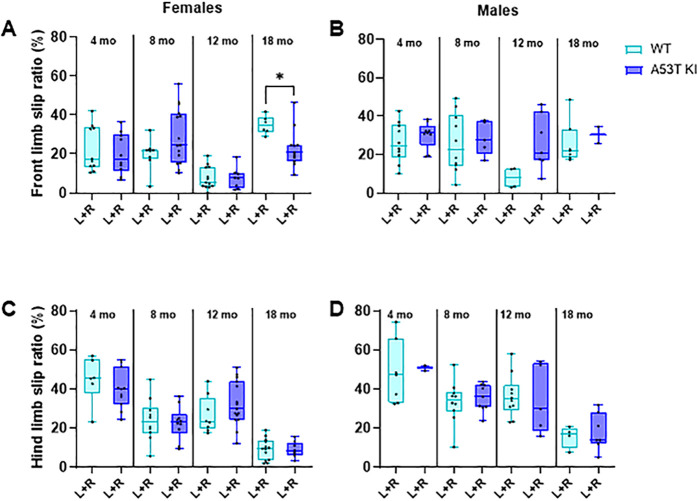
Beam walk test in A53T KI and WT rats reveals minor disturbances in front limbs in males only. Ratio percentage of front limb slips (A, B) and hind limb slips (C, D) in female and male rats. L + R = left + right. Data are presented as box-whisker plots. Data were analyzed using ordinary two-way ANOVA with genotype and age as independent factors. If the genotype × age effect was significant, pairwise comparisons within the same age group were done by the Holm–Šídák’s multiple comparisons test as follows: *P < 0.05.

Additionally, we assessed balance using the beam walk test. Analysis of performance of aSyn A53T KI and WT rats in this test similarly showed that mutant animals had generally unperturbed sensorimotor function (Fig 8). A significant Genotype × Age interaction effect was observed for the front limb slip ratio in female rats (F _(__3, 76__)_ = 4.115; *P* = 0.0092) at 18 months of age (*P =* 0.0268; Holm-Šídák’s multiple comparisons test), however appears to be due to an increase in slips in the WT line rather than motor dysfunction in the A53T aSyn KI line.

**Fig 9:**
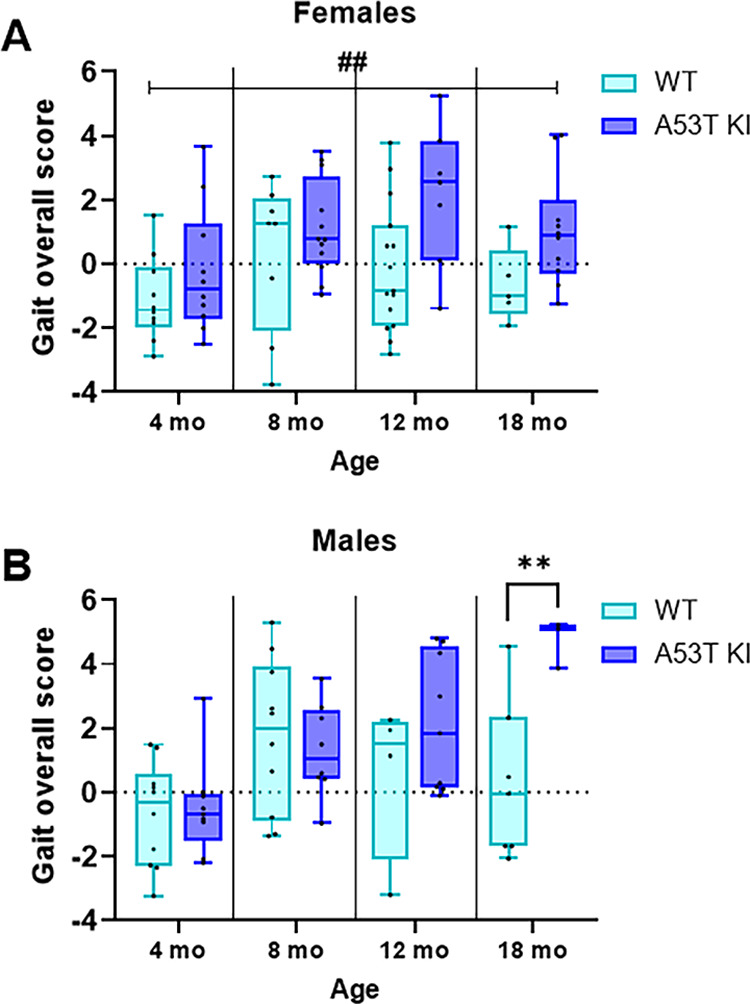
Kinematic analysis of fine motor skills in A53T KI and WT rats. Gait overall score was calculated in 4-, 8-, 12-, and 18-month-old female (A) and male (B) rats as described previously [19]. Ordinary Two-way ANOVA results are indicated as follows: ^##^*P* < 0.01, main genotype effect; **P < 0.01, Holm–Šídák’s multiple comparisons test.

Finally, kinematic analysis of fine motor skills was performed with Principal Component Analysis (PCA) of individual gait parameters measured in the MotoRater apparatus (S4 and S5 Figs). The overall gait score expressed as distance from the pooled score of all WT animals of the corresponding age was influenced by both the genotype and age (Fig 9). In particular, we found that genotype had a significant effect on the gait overall score in both females (F_(1, 69)_ = 10.51; *P* = 0.0018) and males (F_(1, 52)_ = 6.953; *P* = 0.011). Likewise, age had a significant impact on both female (F_(3, 69)_ = 3.082; *P* = 0.033) and male rats (F_(3, 52)_ = 6.297; *P* = 0.001). In addition, the genotype × age interaction had a significant effect on the gait score in male rats (F_(3, 52)_ = 3.281; *P* = 0.028). The Holm–Šídák’s multiple comparisons test indicated that the difference in males was particularly significant between 18-month-old aSyn A53T KI and WT male rats (*P* = 0.0071; Fig 9B). Further dissection of the genotype × age interaction effect in male rats showed that in WT animals, the gait score in 8-month-old (but not 12- or 18-month-old) animals was significantly higher than in 4-month-old animals (*P* = 0.0219, Dunnett’s test). In contrast, in aSyn A53T KI rats, the gait scores in 12- and 18-month-old (but not 8-month-old) animals were significantly higher than in 4-month-old animals (*P* = 0.0187 and *P* = 0.0006, respectively).

### aSyn A53T KI and aSyn KO rats do not display nigrostriatal degeneration

**Fig 10:**
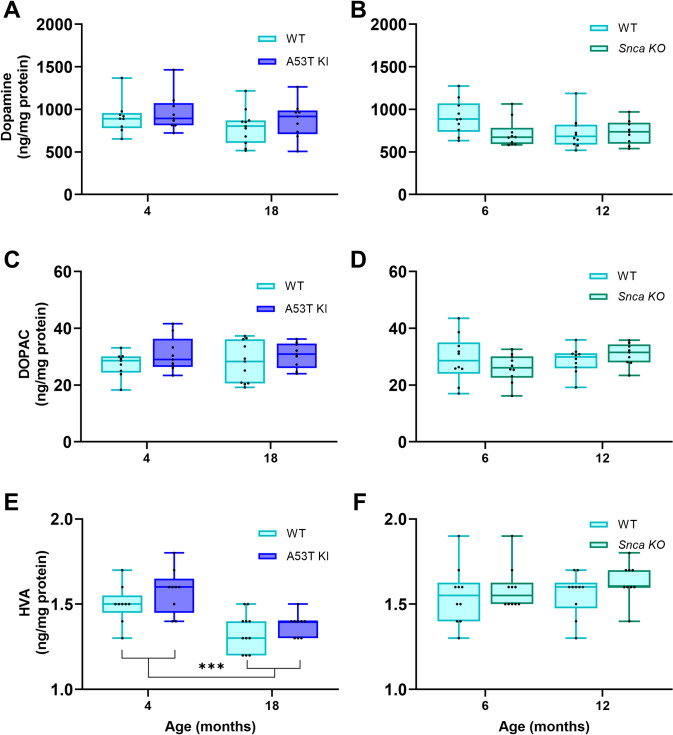
The humanized A53T aSyn KI and endogenous rat aSyn KO do not result in striatal neurochemistry deficits. Striatal levels of dopamine (A, B), DOPAC (C, D), and HVA (E, F) were determined using HPLC in samples of 4- and 18-month-old aSyn A53T KI rats and 6- as well as 12-month-old aSyn KO rats and associated WT littermates. Data were analyzed using Mixed-effects model (REML) with genotype and age as independent factors. Results are indicated as follows: ***P < 0.001 age effect.

To assess degeneration of the nigrostriatal system, we first analyzed striatal dopamine neurochemistry to determine if nigrostriatal terminals were impacted in the aSyn A53T KI or aSyn KO rat lines. HPLC analysis of striatal samples of aSyn A53T KI, aSyn KO, and corresponding WT littermates showed that neither genotype nor the genotype × age interaction affected levels of striatal dopamine or its primary metabolites, 3,4-dihydroxyphenylacetic acid (DOPAC) and homovanillic acid (HVA) in mutant rats (Fig 10). The only statistically significant effect was the overall decrease in HVA content in aSyn A53T KI and WT rats with age (F _(__1, 16__)_ = 34.56, *P* < 0.0001, main effect of age).

**Fig 11:**
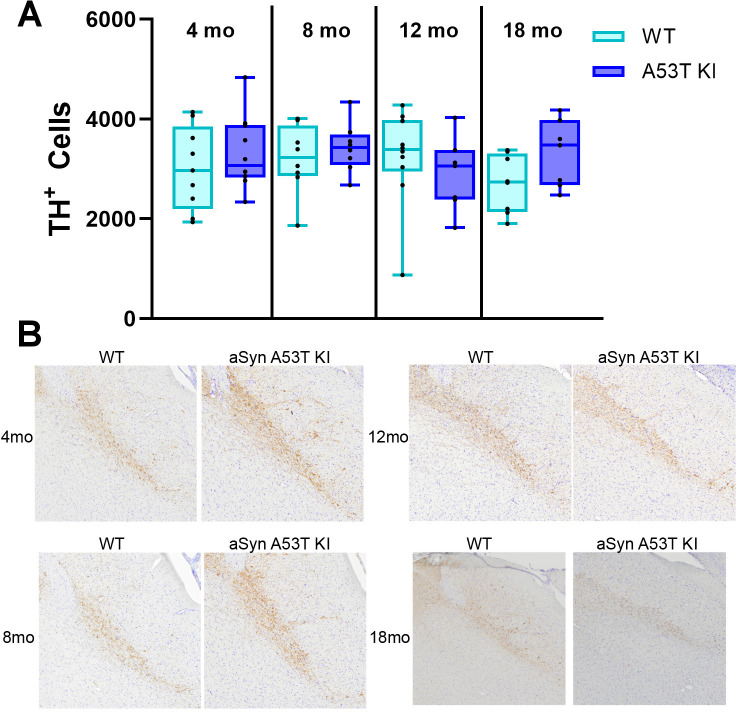
aSyn A53T KI do not display nigral neuron loss. (A) Box-whisker plots of TH^+^ cell numbers in the SNpc of aSyn A53T KI and WT rats. (B) Images of midbrain sections stained for TH to visualize dopaminergic neurons in the SNpc.

Furthermore, we performed immunohistochemical staining for TH in the SNpc to quantify nigral dopamine neurons in the aSyn A53T KI model. Staining and stereology of thin brain sections showed similar numbers of TH^+^ cells in the SNpc of the aSyn A53T KI and WT rats (main effect of genotype: F_(1, 59)_ = 1.197, *P* = 0.278), indicating that the humanized A53T mutation does not spontaneously lead to degeneration of dopaminergic neurons (Fig 11).

### aSyn A53T KI rats do not display gastrointestinal or brain pathology

Although nigrostriatal degeneration and gastrointestinal (GI) dysfunction were not observed in this model, we wanted to confirm if neurodegeneration-related pathology was present in the brain and GI tract. We selected 18–month-old animals for this study, as aSyn, tau, and inflammation-related pathology would be most likely observed in aged animals.

**Fig 12:**
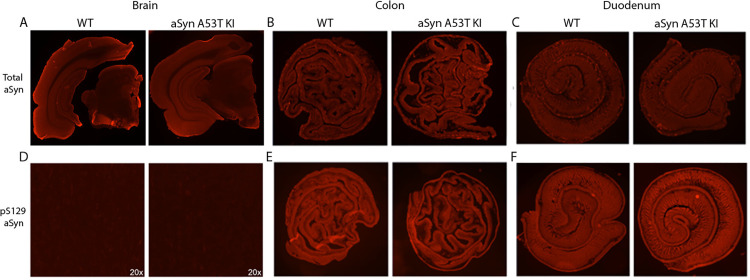
aSyn A53T KI do not display aSyn pathology in the SNpc or gastrointestinal system. (A-C) Representative images of staining for human/rat aSyn in the (A) midbrain, (B) colon, and (C) duodenum of WT and aSyn A53T KI rats. stained for human/rat aSyn. (D-F) Representative images of staining for pS129 aSyn in the (D) SNpc at 20x, (E) colon, and (F) duodenum. Colon and duodenum display high background fluorescence for pS129 aSyn.

**Fig 13:**
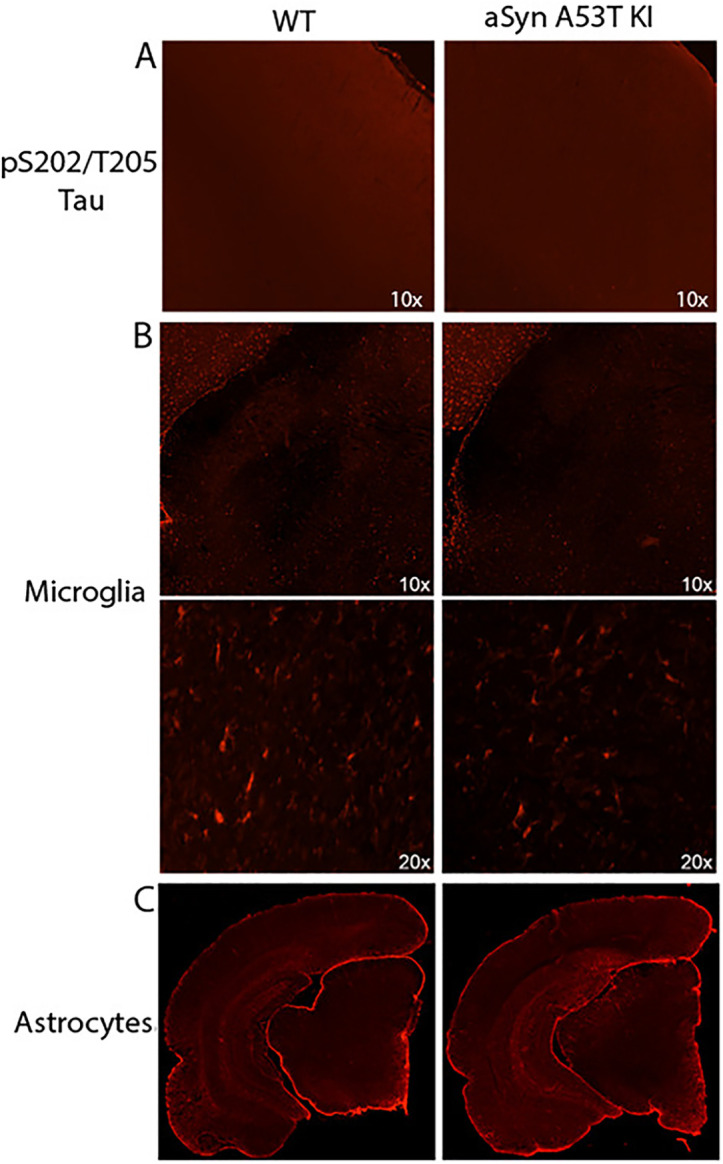
aSyn A53T KI do not display tau pathology or neuroinflammation in brain. (A) Representative images of staining for pS202/T205 tau in the cortex of WT and aSyn A53T KI mice. (B) Representative images of staining for Iba1-positive microglia in the SNpc at 10x and 20x magnification. (C) Representative images of GFAP-positive astrocytes in the brain.

Regarding aSyn expression, we observed similar total aSyn protein levels in the GI tract and brain between aSyn A53T KI rats and WT littermates when using an antibody that recognizes both rat and human aSyn, indicating physiologically-relevant aSyn expression levels in the aSyn A53T KI rat (Fig 12A–C). pS129 aSyn pathology was not observed in the SNpc, colon, or duodenum of aSyn A53T rats (Fig 12D–F). Additional markers of brain pathology--including pS202/T205 tau pathology in the cortex, microgliosis in the substantia nigra, and astrogliosis in the brain--were not observed in the aSyn A53T KI model at 18 months of age (Fig 13).

## Discussion

Alpha-synuclein is linked to Parkinson’s disease through *SNCA* genetic mutations, presence of phosphorylated aSyn in Lewy bodies and Lewy neurites, and evidence of aggregated aSyn in PD patient cerebrospinal fluid. While many rodent models are currently available to study alpha-synuclein in the context of PD, a key gap in the field is a model that expresses human aSyn at physiological levels without the presence of endogenous rodent aSyn. To address this gap, The Michael J. Fox Foundation for Parkinson’s Research developed a novel A53T aSyn rat line using CRISPR/Cas9 to humanize the amino acids surrounding the naturally occurring threonine 53 in the Sprague Dawley rat, resulting in expression of human A53T aSyn without endogenous rat aSyn. Simultaneously, we also developed an aSyn KO Sprague Dawley rat model to confirm that phenotypes/pathology observed were not due to the absence of endogenous rat aSyn. Both models lack expression of rat *Snca* mRNA and aSyn protein (Figs 1–3), with the aSyn A53T KI line successfully expressing human A53T *SNCA* mRNA and aSyn protein throughout the brain (Figs 1 and 3). Furthermore, immunostaining with an antibody that cross-reacts with human and rat aSyn indicates generally equivalent aSyn expression levels between WT and aSyn A53T KI rats (Fig 12), confirming physiological expression levels of human aSyn in this model.

We next evaluated face validity of the aSyn A53T KI rat model by profiling PD-like motor phenotypes, gastrointestinal phenotypes, nigrostriatal degeneration, and various markers of brain pathology to determine whether physiological expression of human A53T aSyn initiated PD-like phenotypes/pathology as occurs in patients [2] and models overexpressing human A53T aSyn [12,13]. We extensively profiled motor behaviors in this model up to 18 months of age given the universal prevalence and progressive nature of motor dysfunction in Parkinson’s disease. We did not observe deficits in open field testing (Fig 5) or the less intrusive homecage behavior monitoring (Fig 6), and the beam walk test remained largely unaffected (Fig 8). While fine motor differences were observed in males at 18 months (Fig 9), the lack of deficits in other motor readouts indicate that this might be a subtle difference that is difficult to measure or detect. It would be prudent to test additional sensitive measures of motor disturbance in 18 month old male aSyn A53T KI rats in the future to determine if this phenotype is robust and reliable.

Regarding non-motor symptoms, gastrointestinal dysfunction, sleep disturbances, and anxiety are common in PD patients [23], with gastrointestinal dysfunction and sleep disturbances appearing prior to motor dysfunction [24–26]. We profiled gastrointestinal function in the aSyn A53T KI rats using a variety of tests and found no evidence of robust changes up to 18 months of age (Fig 4). Evidence of anxiety-like phenotypes can be gleaned from the open field and homecage monitoring tests [27]. Rats did not show overt differences in distance travelled in the center during the open field test (Fig 5C, D), and time spent near the walls in the home cage monitoring was not meaningfully different between aSyn A53T KI rats and WT littermates (Fig 7). Finally, activity patterns during light/dark cycles were similar between aSyn A53T KI rats and WT littermates indicating no overt sleep dysfunction in the model (Figs 6 and 7). It is important to note, however, that these measures of anxiety and sleep are rudimentary and baseline, and more thorough investigation of anxiety or sleep dysfunction using additional tests, technologies, or stressors may uncover phenotypes. Furthermore, additional tests on gastrointestinal function, like whole gut transit time, may uncover phenotypes due to dysfunction in anterior parts of the gut as the methods used here focused more on colonic and lower intestine function.

To determine whether PD-like pathology is present within the brain, we performed a detailed analysis of nigrostriatal integrity at the level of the striatal neuron terminals and nigral cell bodies. Dopamine and dopamine metabolite neurochemistry in the striatum was unchanged in the aSyn A53T KI rat model up to 18 months of age (Fig 10), and nigral TH+ neuron numbers remain unchanged up to 18 months as well (Fig 11). This lack of nigrostriatal degeneration supports the lack of motor dysfunction observed in the model. Furthermore, we examined total aSyn, pS129 aSyn, pS202/T205 Tau, microgliosis, and astrogliosis in the brain through immunostaining and found no evidence of increases or changes in these markers in aSyn A53T KI rats at 18 months of age (Figs 12 and 13). Similarly, total aSyn and pS129 aSyn levels in the colon and duodenum were equivalent between WT and aSyn A53T KI rats (Fig 12).

Taken together, the aSyn A53T KI rat model remains relatively unimpaired up to 18 months of age, with no robust phenotypes related to motor dysfunction, non-motor dysfunction, nigrostriatal degeneration, or brain pathology at baseline. Although this model cannot be recommended as a model of parkinsonism at baseline, we are reassured by these findings as they align with evidence in mouse models that near-physiological expression of human WT or mutant aSyn do not result in robust PD-relevant phenotypes [12]. As this is a humanized knockin model, expression of human aSyn is at physiological levels (Fig 12A–C) and driven by endogenous machinery, therefore making expression levels and patterns more similar to WT than transgenic or BAC/PAC models. This suggests that there are factors outside of the sequence of human aSyn that lead to pathology and Parkinson’s disease in humans. The design of this humanized knockin model enables the introduction of such factors through crosses with other genetically-modified rat models, injection of alpha-synuclein PFF aggregates, or administration of environmental exposures to study the impact of external factors on PD development and pathology driven by human aSyn.

Although the complex etiology of Parkinson’s disease is largely unclear in most cases, environmental risk factors and the propensity of aSyn aggregates to seed the formation of new aggregates in endogenous monomeric protein highlight the importance of investigating external triggers of PD pathogenesis [28,29]. To effectively investigate these triggers and understand their relevance, processes, and possible therapeutic interventions, *in vivo* model systems that express human aSyn are important. For instance, recent structural studies of the aggregated aSyn protein revealed differences between human and mouse aSyn aggregate properties. Specifically, recombinant human and mouse aSyn pre-formed fibrils used in PD seeding models have different structural properties which confer differences in seeding ability. Furthermore, when seeding across species (e.g., human aSyn PFFs in mice or recombinant mouse aSyn) the newly templated aggregates have different properties from the original PFF seeding material. This highlights the importance of using models expressing human aSyn without endogenous rodent aSyn, like the aSyn A53T KI rat, in PFF models designed to study human aSyn aggregate properties or to test therapies directed at human aSyn aggregate structures.

In summary, the aSyn A53T KI rat line is a well-characterized model that does not display PD-like behaviors or pathology up to 18 months of age. As the aSyn A53T KI and aSyn KO rat lines were developed by and are shared openly through The Michael J. Fox Foundation’s Research Tools Program (michaeljfox.org/research-tools), we encourage others to access the lines and investigate their utility in combination with aSyn PFFs, environment stressors, and other dual-hit models. For instance, future studies focusing on viral, toxin, or pollution exposures in this aSyn A53T KI rat model could expose new biological findings on the mechanism by which these risk factors interact with human aSyn and increase the risk for Parkinson’s disease. Furthermore, injecting human aSyn PFFs into the aSyn A53T KI rat to characterize pathology and resulting aSyn aggregate structures would potentially open new, more human-relevant *in vivo* systems for testing therapeutic strategies directed towards aSyn aggregates. These are important research avenues that should be pursued moving forward.

## Supporting information

S1 Fig CRISPR editing design and resulting sequence of the aSyn A53T KI rat. Editing design and humanized sequence in the aSyn A53T KI rat. (A) Visual schematic of the editing design depicting homology arm left (HAL), the targeted exon 4 region and replacement with partial human *SNCA* cDNA, the BGHpA-SV40pA, and the homology arm right (HAR). (B) Sequence alignment comparing the rat aSyn, human aSyn, and the sequence of the human aSyn cDNA incorporated into the model. Yellow highlight indicates the endogenous threonine at amino acid 53 in rat aSyn which was maintained in the aSyn A53T KI line. Pink highlights indicate amino acid differences between rat and human aSyn. Green highlight indicates the amino acids that were altered in rat aSyn through the incorporation of the partial human cDNA. (C) DNA sequencing of donor DNA used for the CRISPR edit, indicating homology arms in black, partial human SNCA cDNA sequence in blue, and BGHpA-SV40pA in orange.
(TIF)

S2 Fig Positive control tissue for pathological marker immunostaining. (A) Representative images of staining for pS202/T205 tau in the cortex of P301S tau transgenic mice. (B) Representative images of staining for pS129 aSyn in the SNpc of LRRK2 G2019S KI mice injected with adeno-associated virus to overexpress human A53T aSyn.
(TIF)

S3 Fig Uncropped western blot images corresponding to Fig 3. Uncropped and unadjusted western blot image of (A) total aSyn and actin control in aSyn KO and WT littermate rats, (B) total aSyn, triton-soluble aSyn, triton-insoluble aSyn and actin control in aSyn A53T KI and WT littermate rats.
(TIF)

S4 Fig Gait characteristics and fine motor skills during walking in female aSyn A53T KI and WT rats. Principal component scores generated by the principal component analysis of analysis of different gait patterns and movements. Movement of different body points in relation to the ground and their correlation in the three spatial dimensions was examined. Data are presented as the mean ± standard error of the mean. Statistical significance of differences is illustrated as follows: ^#^*P* < 0.05; ^##^*P* < 0.01 (Student’s *t*-test). The Correlation heat map depicts the degree of correlation for each walking parameter in the whole data set. Red color means positive correlation and blue means negative correlation. Number in the X-axis represents corresponding PC number.
(TIF)

S5 Fig Gait characteristics and fine motor skills during walking in male aSyn A53T KI and WT rats. Principal component scores generated by the principal component analysis of analysis of different gait patterns and movements. Movement of different body points in relation to the ground and their correlation in the three spatial dimensions was examined. Data are presented as the mean ± standard error of the mean. Statistical significance of differences is illustrated as follows: ^#^*P* < 0.05; ^##^*P* < 0.01 (Student’s *t*-test). The Correlation heat map depicts the degree of correlation for each walking parameter in the whole data set. Red color means positive correlation and blue means negative correlation. Number in the X-axis represents corresponding PC number.
(TIF)
