# Supplementary figures and images for: Expression of human A53T alpha-synuclein without endogenous rat alpha-synuclein fails to elicit Parkinson’s disease-related phenotypes in a novel humanized rat model
Source: PLoS One. 2025 Aug 8;20(8):e0329823. doi: 10.1371/journal.pone.0329823 (PMC12334032; doi:10.1371/journal.pone.0329823)

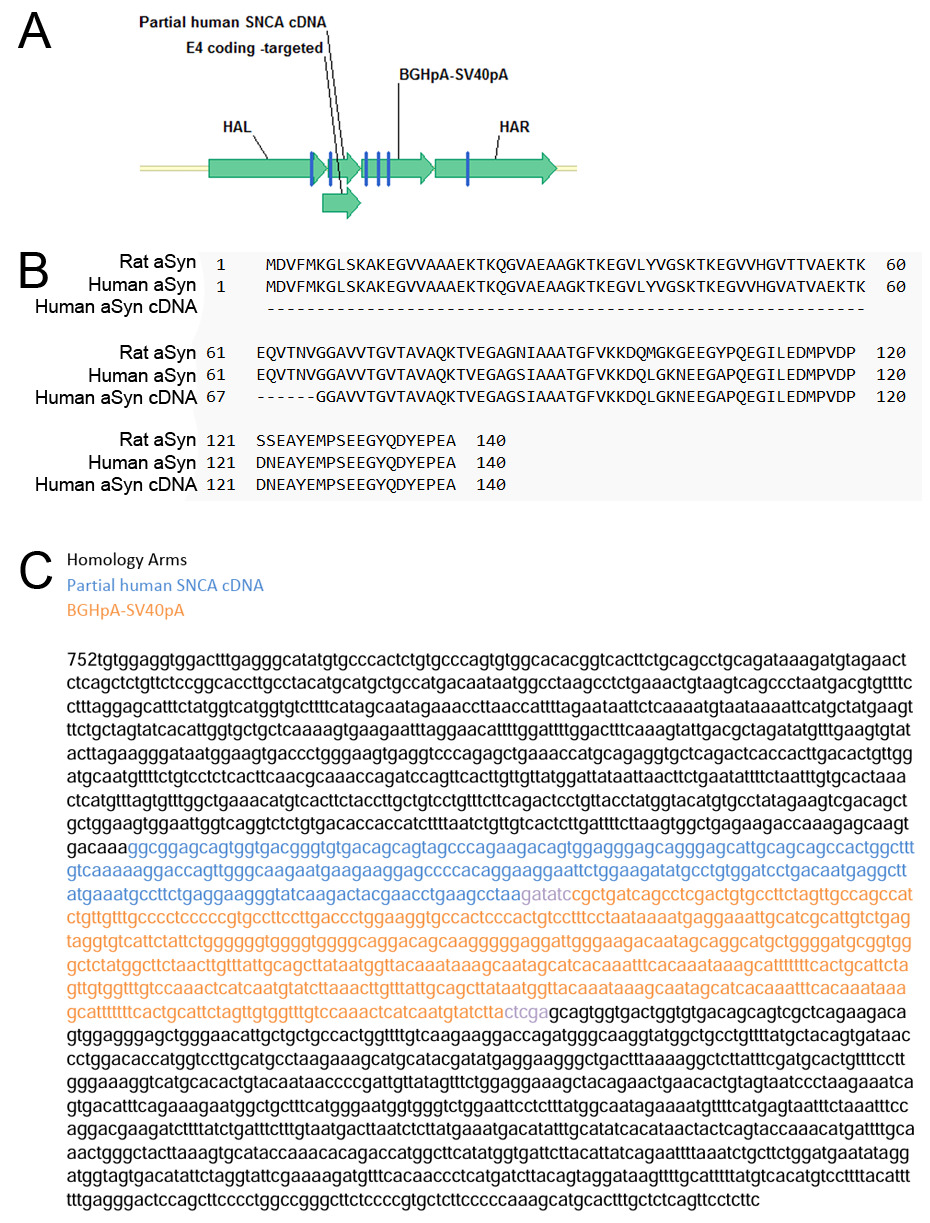

Supplement: S1 Fig — Editing design and humanized sequence in the aSyn A53T KI rat. (A) Visual schematic of the editing design depicting homology arm left (HAL), the targeted exon 4 region and replacement with partial human SNCA cDNA, the BGHpA-SV40pA, and the homology arm right (HAR). (B) Sequence alignment comparing the rat aSyn, human aSyn, and the sequence of the human aSyn cDNA incorporated into the model. Yellow highlight indicates the endogenous threonine at amino acid 53 in rat aSyn which was maintained in the aSyn A53T KI line. Pink highlights indicate amino acid differences between rat and human aSyn. Green highlight indicates the amino acids that were altered in rat aSyn through the incorporation of the partial human cDNA. (C) DNA sequencing of donor DNA used for the CRISPR edit, indicating homology arms in black, partial human SNCA cDNA sequence in blue, and BGHpA-SV40pA in orange. (TIF) [file pone.0329823.s001.tif]

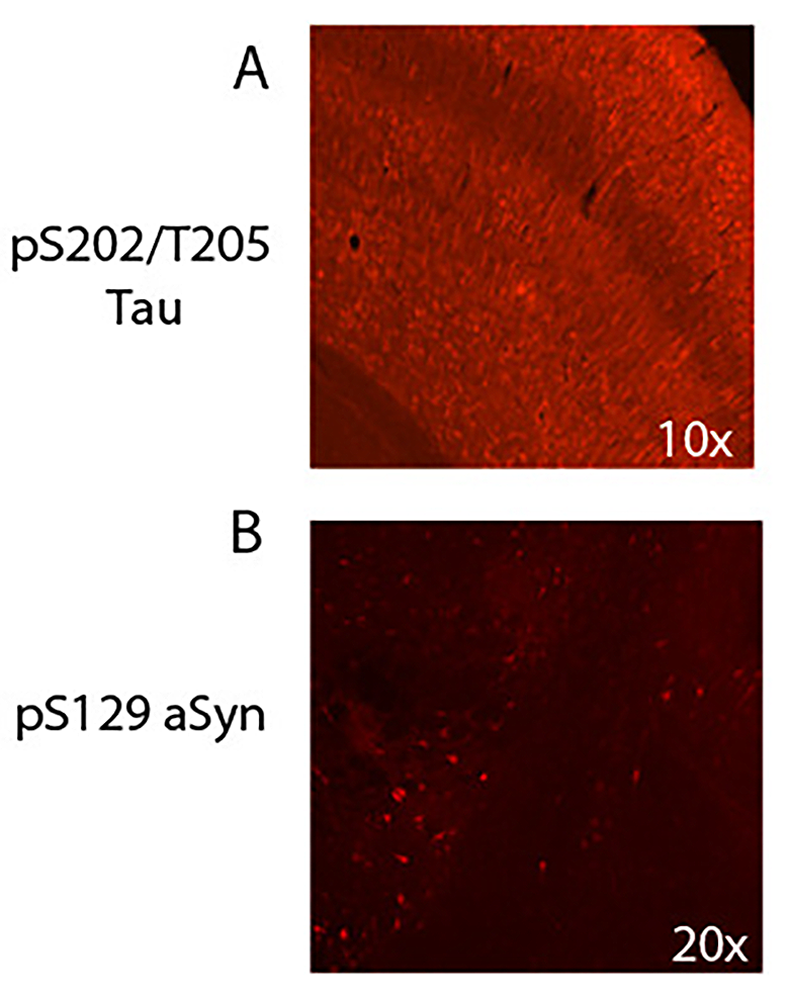

Supplement: S2 Fig — (A) Representative images of staining for pS202/T205 tau in the cortex of P301S tau transgenic mice. (B) Representative images of staining for pS129 aSyn in the SNpc of LRRK2 G2019S KI mice injected with adeno-associated virus to overexpress human A53T aSyn. (TIF) [file pone.0329823.s002.tif]

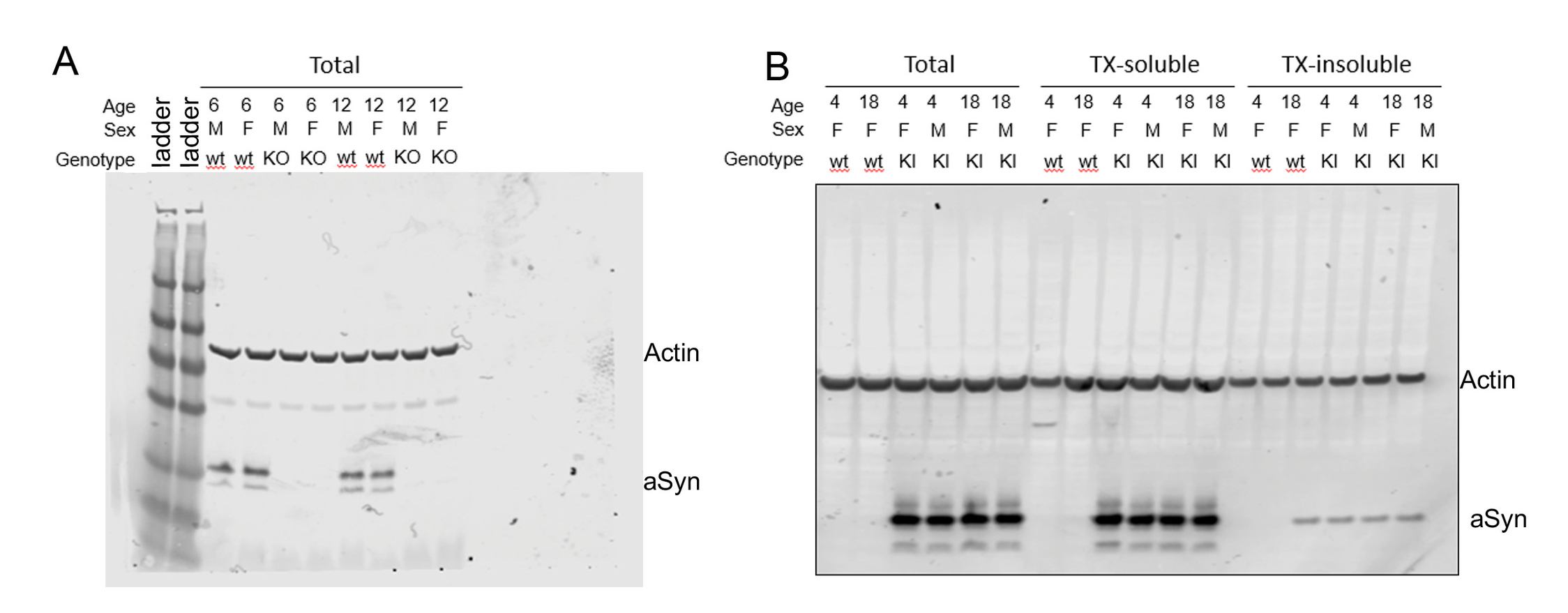

Supplement: S3 Fig — Uncropped and unadjusted western blot image of (A) total aSyn and actin control in aSyn KO and WT littermate rats, (B) total aSyn, triton-soluble aSyn, triton-insoluble aSyn and actin control in aSyn A53T KI and WT littermate rats. (TIF) [file pone.0329823.s003.tif]

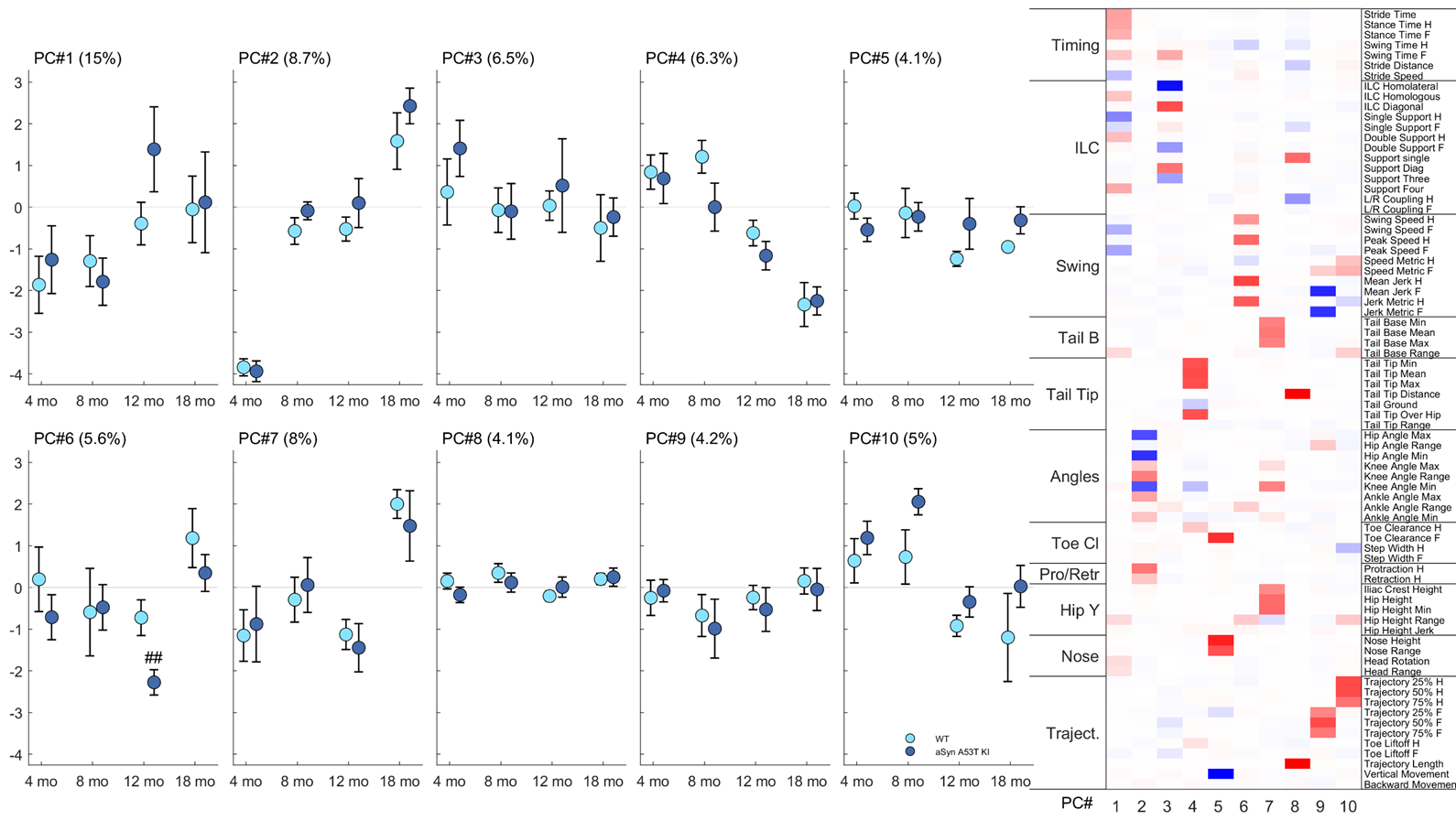

Supplement: S4 Fig — Principal component scores generated by the principal component analysis of analysis of different gait patterns and movements. Movement of different body points in relation to the ground and their correlation in the three spatial dimensions was examined. Data are presented as the mean ± standard error of the mean. Statistical significance of differences is illustrated as follows: #P < 0.05; ##P < 0.01 (Student’s t-test). The Correlation heat map depicts the degree of correlation for each walking parameter in the whole data set. Red color means positive correlation and blue means negative correlation. Number in the X-axis represents corresponding PC number. (TIF) [file pone.0329823.s004.tif]

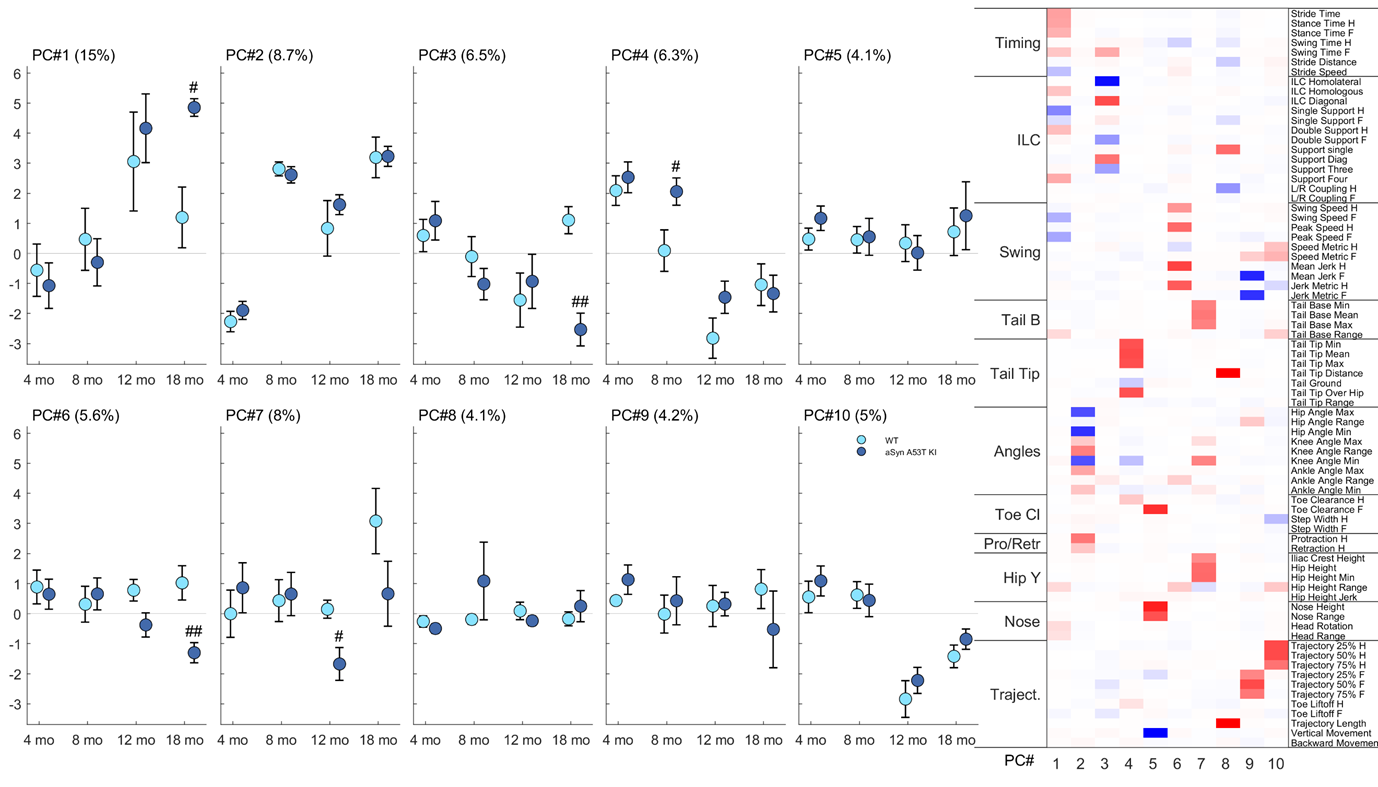

Supplement: S5 Fig — Principal component scores generated by the principal component analysis of analysis of different gait patterns and movements. Movement of different body points in relation to the ground and their correlation in the three spatial dimensions was examined. Data are presented as the mean ± standard error of the mean. Statistical significance of differences is illustrated as follows: #P < 0.05; ##P < 0.01 (Student’s t-test). The Correlation heat map depicts the degree of correlation for each walking parameter in the whole data set. Red color means positive correlation and blue means negative correlation. Number in the X-axis represents corresponding PC number. (TIF) [file pone.0329823.s005.tif]
